# Controlled organocatalyzed d,l-lactide ring-opening polymerizations: synthesis of low molecular weight oligomers†

**DOI:** 10.1039/c8ra05306e

**Published:** 2018-08-14

**Authors:** M. R. Newman, S. G. Russell, D. S. W. Benoit

**Affiliations:** Department of Biomedical Engineering, University of Rochester Rochester NY 14627 USA benoit@bme.rochester.edu; Center for Musculoskeletal Research, University of Rochester Medical Center Rochester NY 14642 USA; Department of Chemical Engineering, University of Rochester Rochester NY 14627 USA

## Abstract

A systematic approach to the synthesis of organocatalyzed oligo(d,l-lactide) demonstrates that choice of initiator, catalytic ratio, and reaction time yields well-controlled oligomers. Ring-opening polymerization of d,l-lactide with the initiator α-methyl propargyl alcohol, a secondary alcohol, used in excess of 4-dimethylaminopyridine catalyst mitigates cyclicization, transesterification, and catalyst-initiated side reactions. This approach enables the design of uniform lactide oligomers for controlled release applications, such as delivery systems for drugs, prodrugs, and molecular sensors.

## Abstract 2

A systematic approach to the synthesis of organocatalyzed oligo(d,l-lactide) demonstrates that choice of initiator, catalytic ratio, and reaction time yields well-controlled oligomers.

## Graphical abstract

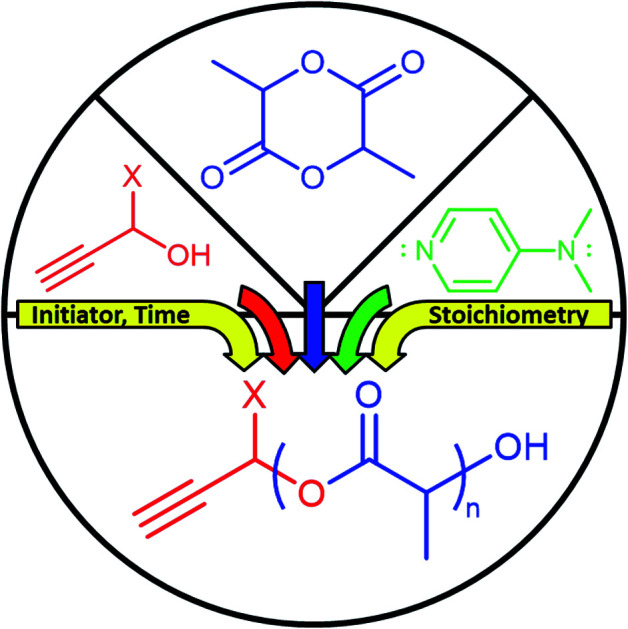

Poly(lactide) (PLA) is a versatile polymer with properties suitable for a range of controlled release applications. Molecular weight and copolymer composition control degradation and drug release, with degradation yielding biocompatible lactic acid products. PLA and copolymers of lactide and glycolide (PLGA) have been used pre-clinically and clinically for release of drugs,^1–3^ prodrugs,^4–6^ and molecular sensors.^7–9^ Highly reproducible PLA chemistries are particularly important to ensure control over the release of drugs with narrow therapeutic windows and to enhance efficacy by reducing dependence on patient compliance.^10–13^ Stannous octoate is commonly employed as an organometallic catalyst of lactide ring-opening polymerization (ROP).^14,15^ However, tin catalysts, which are challenging to fully remove during purification, can result in toxicity.^16^ Alternatives such as strongly basic amine organocatalysts are favored, particularly 4-dimethylaminopyridine (DMAP), which was pioneered by Nederberg *et al.*^17^ DMAP-mediated ROP is used for one-pot PLA polymerizations and conjugations,^18^ diblock or triblock copolymerizations with lactide as a first or second block,^19–21^ grafting lactide to cellulose polymer fibers,^22^ and synthesizing star-shaped/cross-linked PLA networks.^23^ PLA has also been exploited to tether and release drugs from linear polymers and hydrogel depots.^24^ Drug tethers are typically low molecular weight oligo(lactide) composed of fewer than seven lactide repeat units to avoid crystallinity and associated challenges with solubility and control over degradation rates.^25^ Self-catalysis, or direct polycondensation, of lactic acid at increased temperature and reduced pressure for an extended time yields low-molecular weight (800–3200 Da) oligo(lactide), in contrast to organocatalysts that are commonly employed for high molecular weight PLA synthesis. However, extensive setup and environmental control decrease the accessibility of these reactions.^26,27^ Furthermore, with neither an initiator nor a catalyst used in polycondensation, mixtures of α-hydroxy and ω-carboxy PLA are formed, limiting the versatility of post-polymerization drug functionalization.^28^ Alternatively, click chemistry^29^ may be exploited to control subsequent modification of oligo(lactide) by employing a ‘clickable’ alcohol to initiate oligo(lactide) synthesis.^30^

Here, we explored bulk ROP of d,l-lactide by propargyl alcohol initiator and DMAP catalyst to synthesize low molecular weight oligo(lactide) linkers. To enable subsequent click reactions and to mitigate crystallinity, propargyl alcohol (PA) and d,l-lactide (L) were used.^27^ Although not studied here, similar approaches have shown products do not epimerize, and we expect oligomers to be atactic and amorphous.^17^ To investigate the molecular weight and polydispersity of oligo(lactide), an initial polymerization was designed similar to that of Nederberg *et al.*^17^ and Coulembier and Dubois^18^ using a PA : L : DMAP ratio of 1 : 20 : 4 (Scheme 1; see Table S1, ESI†). The neat polymerization was stirred at 130 °C under a nitrogen environment. After 5, 10, 15, 30, and 60 minutes, reaction vials were opened to atmosphere and cooled before dissolution in dichloromethane (DCM) and precipitation in hexanes. ^1^H-NMR spectroscopy identified successful synthesis of PA-functional oligo(d,l-lactide) (PA-ODLA) with ∼99% conversion of d,l-lactide after only 5 minutes of polymerization (Fig. 1a; see Fig. S3, ESI†). Integration of peaks C, E, and F indicated linkers were ∼19 lactide units, or an average *M*_n_ of 2825 Da. However, matrix assisted laser desorption ionization time of flight mass spectrometry suggested *M*_n_ was 752 Da. As *M*_n_ determined by NMR was based on average end-group analysis and assumed PA-initiated oligo(lactide), and *M*_n_ determined by MALDI analysis represented all species present, these data indicated that not all oligo(lactide) chains were initiated by PA. Rather, distinct series of peaks periodically separated by 144 Da, the *M*_n_ of lactide, were formed during polymerization (α, β, γ, δ, ε, and ζ; Fig. 1b; see Table S2, ESI†). Peaks 72 Da less than these peaks were also identified (α′–ζ′). Additional reactions were conducted to identify the formed products: one with the initiator α-methyl propargyl alcohol (αMPA; see Fig. S4, ESI†), and another with only d,l-lactide and DMAP but no initiator (see Fig. S9, ESI†).

**Scheme 1:**
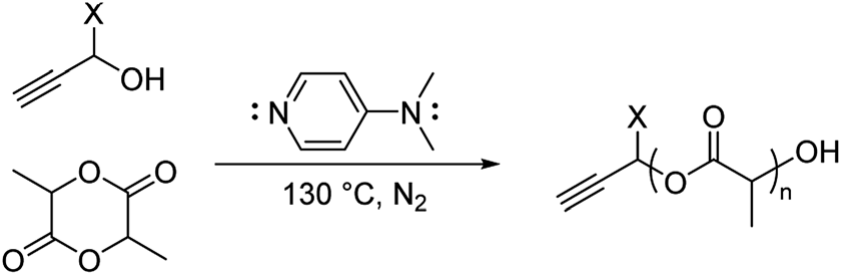
Ring-opening polymerization of lactide by alcohol initiator. X = H, propargyl alcohol; CH_3_, α-methyl propargyl alcohol.

**Fig. 1:**
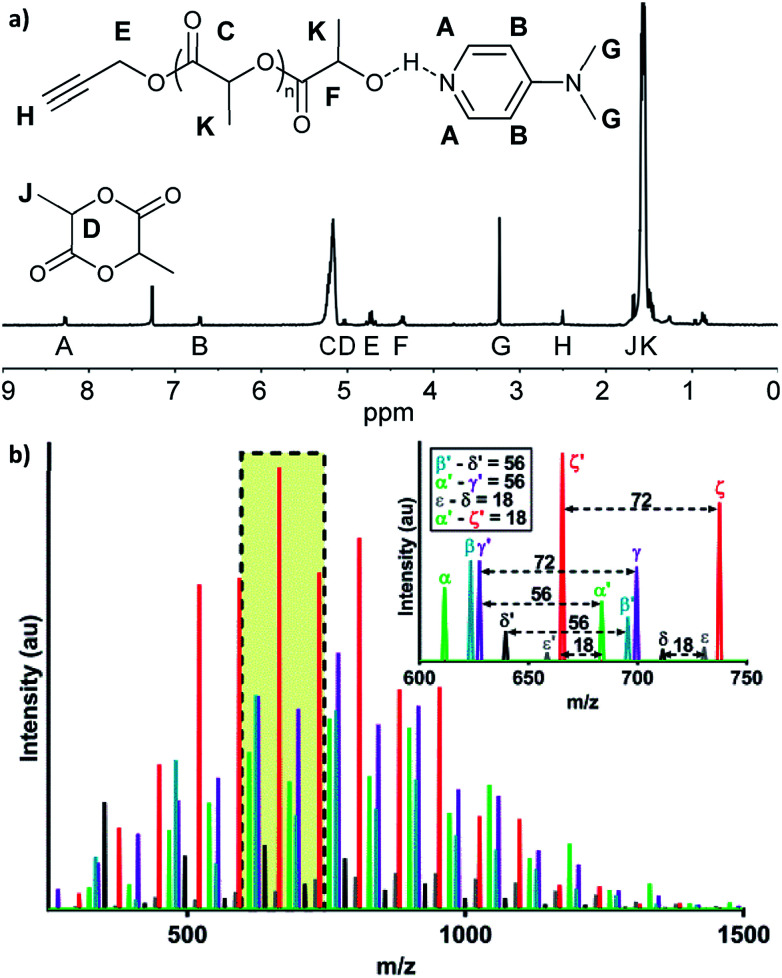
Five minute polymerizations of 1 : 20 : 4 propargyl alcohol : lactide : 4-dimethylaminopyridine form varied products. (a) ^1^H-NMR, with hydrogen peaks assignments shown. (b) MALDI-TOF, where colors represent peak sets that repeat every 72 Da and the inset shows a full 144 Da peak set.

The desired product, PA-ODLA “α” (Fig. 1a), was formed *via* DMAP base activation of PA, which initiated ROP (see Fig. S5, ESI†). Base activation of the alcohol was confirmed similar to previous reports^20,31^ using ^1^H-NMR of PA mixed with DMAP in CDCl_3_ to identify ppm shifts for hydroxyl groups (see Fig. S6, ESI†). Peak α′, 72 Da less than α, was the result of transesterification of PA-ODLA (see Fig. S7, ESI†), which is a common side-reaction during ROP of lactide.^18,20^ Two undesired products, peaks ζ and ζ′, were cyclic PA-ODLA with and without transesterification, respectively. Cyclicization increases over time during ROP of lactide^32–34^ and is undesired because hydroxyl end groups are not available for subsequent conjugation. These peaks were due to radical-mediated dehydration with hydroxyl end group participation,^35^ as the addition of hydroquinone (HQ), a radical scavenger, eliminated peaks ζ and ζ′ (see Fig. S8, ESI†). However, a peak appeared that corresponded to HQ-DMAP-ODLA and lacked alkyne functionalities for subsequent click reactions.

Another undesired product, DMAP-ODLA, or peak γ, formed *via* DMAP nucleophilic attack of lactide (see Fig. S9, ESI†). Peak γ′, separated by 72 Da from peak γ, resulted from transesterification of DMAP-ODLA. By conducting reactions of only DMAP and lactide, only PA and lactide, and only αMPA and lactide, it was confirmed that DMAP can both initiate and catalyze lactide ROP, but PA and αMPA cannot (see Fig. S10, ESI†). DMAP-ODLA formed due to PA : DMAP ratios less than 1, as previously described,^18^ and is undesired because ‘clickable’ propargyl end groups are not present. Finally, peaks β, δ, and ε are likely ion fragments, as these peaks were only present when using MALDI in linear, but not reflector, mode (see Fig. S11, ESI†).

To increase the amount of oligo(lactide) with alkyne and hydroxyl functionalities, side reactions were systematically addressed. It was noted that transesterification increased with time of polymerization and was greater for PA than for αMPA, as less nucleophilic secondary alcohols are unable to participate in transesterification reactions.^30^ Thus, reactions were conducted at 130 °C for 5 minutes using αMPA as the initiator to optimize desired product (αMPA-ODLA, peak α).

Ratios of αMPA : L : DMAP were investigated to mitigate undesired DMAP-initiated and cyclic ODLA (Table 1). Holding αMPA : L constant and increasing αMPA : DMAP increased the intensity of cyclic αMPA-ODLA ζ relative to DMAP-ODLA γ, but αMPA-ODLA α became negligible (Fig. 2a; see Fig. S12, ESI†). Increasing αMPA : L at a constant αMPA : DMAP increased the intensity of α relative to both ζ and γ (Fig. 2b; see Fig. S13, ESI†). Similar behavior was identified when holding L : DMAP constant and increasing αMPA : L (Fig. 2c; see Fig. S14, ESI†). These results suggest that αMPA : L controls cyclicization, as the distribution of ζ is similar among reactions in Fig. 2a, and the ratio of ζ : α decreases between ratios of 1 : 10 and 1 : 2 in Fig. 2b. αMPA : DMAP controls DMAP-ODLA generation, as the distribution of γ is similar within Fig. 2b and smallest at a ratio of 2 : 1. Finally, lower ratios of L : DMAP appear to increase α, with 2 : 1 yielding the greatest amount of desired product α. Altogether, higher αMPA : L, higher αMPA : DMAP, and lower L : DMAP ratios yield higher levels of α relative to γ and ζ.

**Table 1:** Characterization of 5 minute αMPA polymerizations. *X* = % conversion, *M*_n_ = molecular weight, PDI = polydispersity

αMPA : L : DMAP	*X*	*M*_n_ (NMR)	*M*_n_ (MALDI)	PDI
1 : 20 : 4	96%	2040 Da	721 Da	1.16
1 : 20 : 2	87%	3620 Da	782 Da	1.20
1 : 20 : 1	88%	3150 Da	826 Da	1.23
1 : 20 : 0.5	65%	3120 Da	881 Da	1.23
1 : 10 : 1	92%	2200 Da	759 Da	1.25
1 : 5 : 1	97%	1470 Da	763 Da	1.16
1 : 2 : 1	97%	1160 Da	656 Da	1.10
1 : 10 : 5	96%	2050 Da	772 Da	1.11
1 : 5 : 2.5	97%	1550 Da	725 Da	1.12

**Fig. 2:**
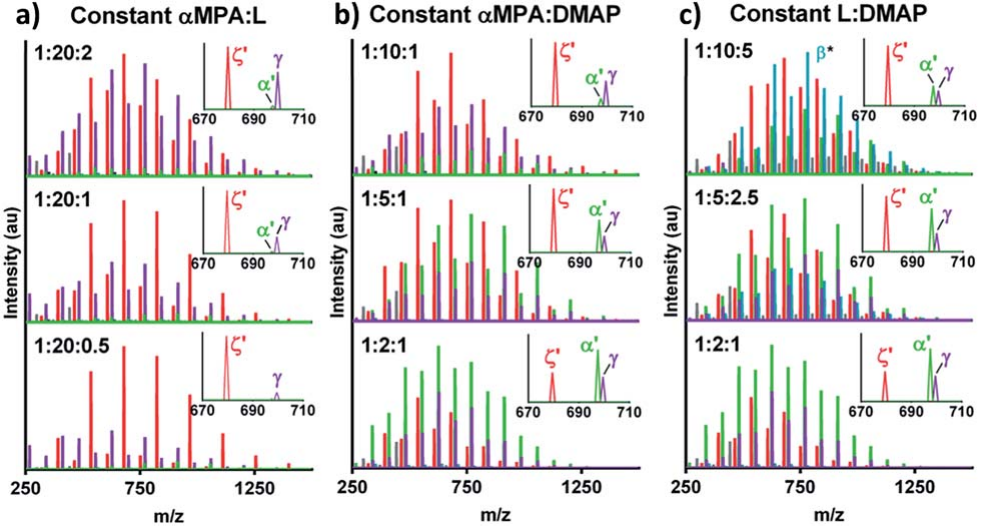
Initiator, catalyst, and monomer relationships control ratios of formed products. (a) MALDI-TOF with (top to bottom) increasing αMPA : DMAP and L : DMAP. (b) MALDI-TOF with increasing αMPA : L and decreasing L : DMAP. (c) MALDI-TOF with increasing αMPA : DMAP and αMPA : L. *β is a temporary ion fragment (see Fig. 1).

Increasing the stoichiometry of lactide to initiator is commonly exploited to form linkers of various lengths to achieve differential release rates.^24^ To explore the utility of this approach using optimized reaction conditions, αMPA : L ratios of 1 : 2, 1 : 5, and 1 : 10 were investigated using 1 mol% DMAP (Table 2, see Fig. S15–S17, ESI†). With an αMPA : DMAP ratio of 33 or greater, γ was absent in all reactions. The polymerization with the highest αMPA : L ratio, 1 : 2 : 0.03, had the greatest overall α : ζ ratio. Interestingly, the peak η appeared, corresponding to αMPA-ODLA with a K^+^ ion, in contrast to α with a DMAP-H^+^ ion adduct. There was a transition from η to α over time, and η was most abundant in 1 : 2 : 0.03 reactions. This was explored further with polymerization times of 4 minutes and less (see Fig. S18, ESI†). One minute reactions showed negligible conversion of lactide monomer. After 2 minutes, conversion was 68% and the major product was η. By 4 minutes, α and ζ exceeded η, suggesting that shorter reaction times are necessary to isolate linear αMPA-ODLA without DMAP adducts.

**Table 2:** Characterization of αMPA-initiated reactions of various stoichiometry and reaction times. *X* = % conversion, *M*_n_ = molecular weight

αMPA : L : DMAP	Time	*X*	α : ζ	*M*_n_ (MALDI)
1 : 2 : 0.03	5 min	82%	1.1	619 Da
	10 min	99%	1.3	685 Da
	15 min	99%	1.6	661 Da
1 : 5 : 0.06	5 min	73%	0.13	753 Da
	10 min	89%	0.23	727 Da
	15 min	94%	0.24	758 Da
1 : 10 : 0.11	5 min	32%	0.25	772 Da
	10 min	71%	0.10	736 Da
	15 min	83%	0.08	747 Da

Finally, proof of concept conjugations demonstrated the utility of αMPA-ODLA as a heterobifunctional linker (Fig. 3; see Fig. S19, ESI†). Following 90 seconds of polymerization of 1 : 2 : 0.03 αMPA : L : DMAP (Fig. 3a), αMPA-ODLA was modified on either propargyl or hydroxyl end groups. 3-Azido-1-propanol (see Fig. S20, ESI†) was added to propargyl moieties *via* Huisgen 1,3-dipolar cycloaddition (Fig. 3b). Interestingly, only η was shifted by 101 Da, the *M*_n_ of 3-azido-1-propanol, by MALDI analysis, suggesting that DMAP adducts in α interfere with propargyl modification. Hydroxyl end groups were modified to carboxylic acids using two-step, one-pot reactions whereby succinic anhydride (SA) was added following 90 seconds of lactide polymerization (Fig. 3c). These reactions were viable in either order when modifying both ends of αMPA-ODLA (Fig. 3d, S19d and e, see ESI†). It is noted that since an alcohol was used as a model azide, both N_3_-αMPA-ODLA-SA and SA-N_3_-αMPA-ODLA-SA formed during reactions. Pure N_3_-αMPA-ODLA-SA could be achieved by forming αMPA-ODLA-SA, removing unreacted SA, and conducting azide–alkyne conjugation.

**Fig. 3:**
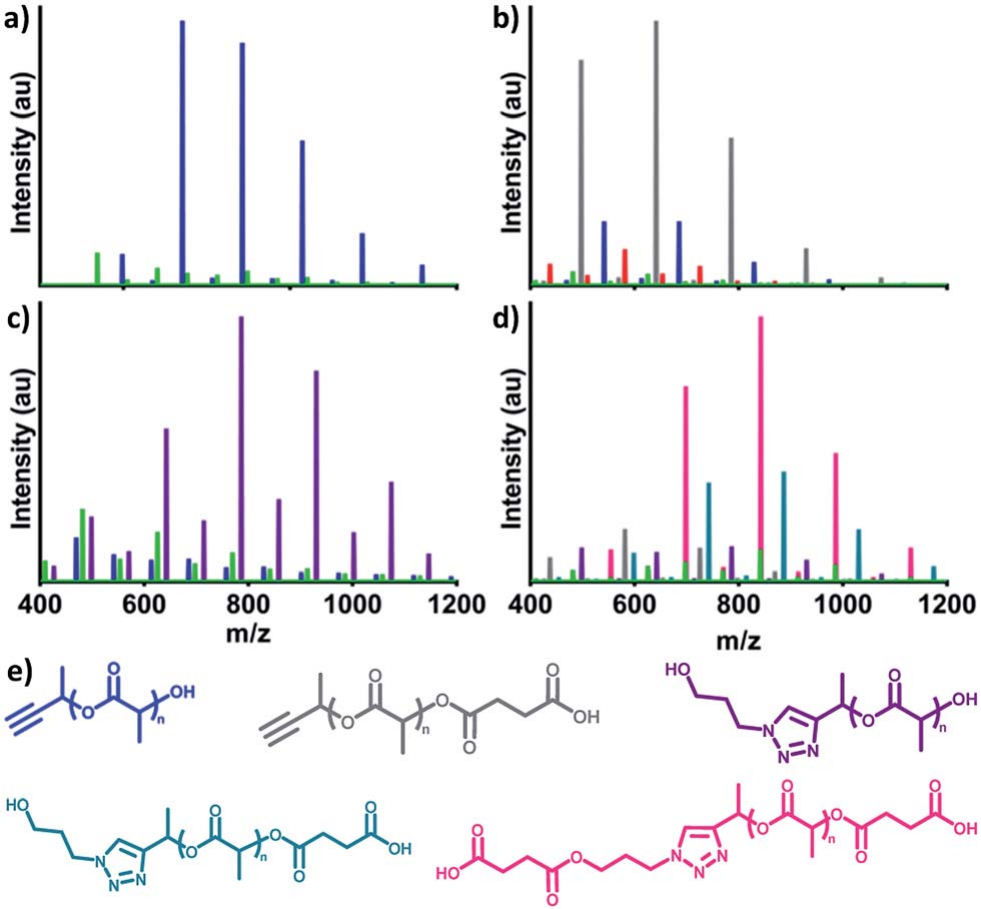
Demonstration of αMPA-ODLA linker heterobifunctionality using MALDI-TOF analysis. (a) Polymerization to form αMPA-ODLA. (b) Reaction to open succinic anhydride (SA) on αMPA-ODLA. (c) Reaction to add 3-azido-1-propanol (N_3_) on αMPA-ODLA. (d) Reaction to add 3-azido-1-propanol on αMPA-ODLA-SA. (e) Chemical structures of αMPA-ODLA: blue, αMPA-ODLA-SA: grey, N_3_-αMPA-ODLA: purple, N_3_-αMPA-ODLA-SA: teal, SA-N_3_-αMPA-ODLA-SA: pink.

## Conclusions

This work presents a systematic design of narrowly dispersed, low molecular weight oligo(lactide) formed without metal catalysts that are difficult to completely remove during purification. Before optimization, observed MALDI *M*_n_ disagreed with *M*_n_ as determined by NMR, suggesting transesterification reactions were present. Previous studies investigated the singular effects of polymerization temperature, polymerization time, monomer : initiator ratio, chemical initiator, and reagent degassing time on PLA transesterification.^36^ This study identified the combined effects of choice of initiator, initiator : catalyst ratio, initiator : monomer ratio, and polymerization time on molecular weight, polydispersity, and relative amounts of desired and undesired products. Temperature was not changed from 130 °C due to the high melting point of d,l-lactide (122 °C). A 90 second reaction of 1 : 2 : 0.03 αMPA : L : DMAP formed oligo(lactide) with alkyne and hydroxyl functionalities available for subsequent click conjugations. Further optimization may be required to increase the reaction efficiency, as theoretical *M*_n_ does not match *M*_n_ determined by MALDI. Although studies herein investigated ROP of lactide by DMAP, the approach can be employed for similar systems (*e.g.* catalysis by stannous octoate or ROP of glycolide). Similarly, copper-catalyzed azide–alkyne cycloaddition (CuAAC) may be used in place of Huisgen 1,3-dipolar cycloaddition for applications in which residual copper contamination is acceptable.

## Conflicts of interest

There are no conflicts to declare.

## Supplementary Material

RA-008-C8RA05306E-s001

## References

[cit1] Guan C., Xu B., Qiao S., Qin L., Li Y., Li Z., Guo Y., Sun Z., Song L., Gao R., Investigators P. I. (2017). Cathet. Cardiovasc. Interv..

[cit2] Salama A. H., Mahmoud A. A., Kamel R. (2016). AAPS PharmSciTech.

[cit3] Gholizadeh S., Kamps J., Hennink W. E., Kok R. J. (2017). Int. J. Pharm.

[cit4] Ren K., Zhang M., He J., Wu Y., Ni P. (2015). ACS Appl. Mater. Interfaces.

[cit5] Veurink M., Asmus L., Hennig M., Kaufmann B., Bagnewski L., Heiligenhaus A., Mendrinos E., Pournaras C. J., Gurny R., Moller M. (2013). Eur. J. Pharm. Sci..

[cit6] Sobczak M., Witkowska E., Oledzka E., Kolodziejski W. (2008). Molecules.

[cit7] Jaidev L. R., Chellappan D. R., Bhavsar D. V., Ranganathan R., Sivanantham B., Subramanian A., Sharma U., Jagannathan N. R., Krishnan U. M., Sethuraman S. (2017). Acta Biomater..

[cit8] Lin W., Li Y., Zhang W., Liu S., Xie Z., Jing X. (2016). ACS Appl. Mater. Interfaces.

[cit9] Zhang Q., Du Y., Jing L., Liang X., Li Y., Li X., Dai Z., Tian J. (2016). J. Biomed. Nanotechnol..

[cit10] Xie J., Li A., Li J. (2017). Macromol. Rapid Commun..

[cit11] Leahy L. G. (2017). J. Psychosoc. Nurs. Ment. Health Serv..

[cit12] Fan Y. L., Hou H. W., Tay H. M., Guo W. M., Berggren P. O., Loo S. C. (2017). AAPS PharmSciTech.

[cit13] Rafiei P., Haddadi A. (2017). Pharm. Nanotechnol..

[cit14] Kaihara S., Matsumura S., Mikos A. G., Fisher J. P. (2007). Nat. Protoc..

[cit15] Leenslag J. W., Pennings A. J. (1987). Makromol. Chem..

[cit16] Tanzi M. C., Verderio P., Lampugnani M. G., Resnati M., Dejana E., Sturani E. (1994). J. Mater. Sci.: Mater. Med..

[cit17] Nederberg F., Connor E. F., Moller M., Glauser T., Hedrick J. L. (2001). Angew. Chem., Int. Ed. Engl..

[cit18] Coulembier O., Dubois P. (2012). J. Polym. Sci., Part A: Polym. Chem..

[cit19] Makiguchi K., Kikuchi S., Yanai K., Ogasawara Y., Sato S., Satoh T., Kakuchi T. (2014). J. Polym. Sci., Part A: Polym. Chem..

[cit20] Kadota J., Pavlovic D., Hirano H., Okada A., Agari Y., Bibal B., Deffieux A., Peruch F. (2014). RSC Adv..

[cit21] Dimitrov I. V., Berlinova I. V., Michailova V. I. (2013). Polym. J..

[cit22] Yan C. H., Zhang J. M., Lv Y. X., Yu J., Wu J., Zhang J., He J. S. (2009). Biomacromolecules.

[cit23] Eldessouki M., Buschle-Diller G., Gowayed Y. (2016). Des. Monomers Polym..

[cit24] Benoit D. S. W., Nuttelman C. R., Collins S. D., Anseth K. S. (2006). Biomaterials.

[cit25] de Jong S. J., De Smedt S. C., Demeester J., van Nostrum C. F., Kettenes-van den Bosch J. J., Hennink W. E. (2001). J. Controlled Release.

[cit26] Proikakis C. S., Tarantili P. A., Andreopoulos A. G. (2002). J. Elastomers Plast..

[cit27] Fukuzaki H., Yoshida M., Asano M., Kumakura M. (1989). Eur. Polym. J..

[cit28] Liu Y., Hou W., Sun H., Cui C., Zhang L., Jiang Y., Wu Y., Wang Y., Li J., Sumerlin B. S., Liu Q., Tan W. (2017). Chem. Sci..

[cit29] Kolb H. C., Finn M. G., Sharpless K. B. (2001). Angew. Chem., Int. Ed. Engl..

[cit30] Nederberg F., Connor E. F., Glausser T., Hedrick J. L. (2001). Chem. Commun..

[cit31] Bonduelle C., Martin-Vaca B., Cossio F. P., Bourissou D. (2008). Chemistry.

[cit32] Kricheldorf H. R., Weidner S. M., Scheliga F. (2017). Polym. Chem..

[cit33] Katiyar V., Nanavati H. (2010). Polym. Chem..

[cit34] Montaudo G., Montaudo M. S., Puglisi C., Samperi F., Spassky N., LeBorgne A., Wisniewski M. (1996). Macromolecules.

[cit35] Lipik V. T., Widjaja L. K., Liow S. S., Abadie M. J. M., Venkatraman S. S. (2010). Polym. Degrad. Stab..

[cit36] Schwach G., Coudane J., Engel R., Vert M. (2002). Biomaterials.

