# Supplementary material for: Controlled organocatalyzed d,l-lactide ring-opening polymerizations: synthesis of low molecular weight oligomers
Source: RSC Adv. 2018 Aug 14;8(51):28891–4. doi: 10.1039/c8ra05306e (PMC9084349; doi:10.1039/c8ra05306e)
Supplement: RA-008-C8RA05306E-s001 [file RA-008-C8RA05306E-s001.pdf]

Electronic Supplementary Material (ESI) for RSC Advances.
This journal is © The Royal Society of Chemistry 2018

# Controlled organocatalyzed D,L-lactide ring-opening polymerizations: synthesis of low molecular weight oligomers

M. R. Newman,[a-b] S. G. Russell,[c] and D. S. W. Benoit[a-c†]

[a.] *Department of Biomedical Engineering, University of Rochester, Rochester, NY 14627 USA.*
[b.] *Center for Musculoskeletal Research, University of Rochester Medical Center, Rochester, NY 14642 USA.*
[c.] *Department of Chemical Engineering, University of Rochester, Rochester, NY 14627 USA.*
† Corresponding author: benoit@bme.rochester.edu.

## Electronic Supplementary Information

## Contents

Experimental ..... 3
Reagents and equipment ..... 3
General Synthesis ..... 3
Hydroxyl end group modification ..... 3
Huisgen 1,3-dipolar cycloaddition ..... 3
$^1$H-NMR ..... 4
Matrix assisted laser desorption ionization time of flight (MALDI-TOF) ..... 4
Peak designation by MALDI-TOF analysis ..... 4
Figures and Tables ..... 5
Figure S1. ..... 5
Figure S2. ..... 6
Table S1. ..... 7
Figure S3a. ..... 8
Figure S3b-c. ..... 9
Figure S4a. ..... 10
Figure S4b-c. ..... 11
Table S2. ..... 12
Figure S5. ..... 12
Figure S6. ..... 13
Figure S7. ..... 14
Figure S8. ..... 14

Figure S9. ....................................................................................................................................15
Figure S10. ..................................................................................................................................16
Figure S11. ..................................................................................................................................17
Figure S12. ..................................................................................................................................18
Figure S13. ..................................................................................................................................19
Figure S14. ..................................................................................................................................20
Figure S15. ..................................................................................................................................21
Figure S16. ..................................................................................................................................22
Figure S17. ..................................................................................................................................23
Figure S18. ..................................................................................................................................24
Figure S19. ..................................................................................................................................25
Figure S20. ..................................................................................................................................26

## Experimental

### Reagents and equipment

All reagents were ≥98% pure and used as received. Propargyl alcohol (2-Propyn-1-ol), α-methyl propargyl alcohol ((±)-But-3-yn-2-ol), D,L-lactide (3,6-Dimethyl-1,4-dioxane-2,5-dione), 4-dimethylaminopyridine (DMAP), succinic anhydride (SA), trifluoroacetic acid (TFA), and 3-chloro-1-propanol were purchased from Alfa Aesar. Hydroquinone was purchased from Sigma Aldrich. Dichloromethane (DCM), hexanes, and acetonitrile, *N,N*-dimethylformamide (DMF), and diethyl ether were purchased from Fisher Scientific. Deionized distilled water ($ddH_2O$) with a resistivity of 18.2 MΩ/cm was obtained using a Barnstead ultrapure water filtration system. Alpha-Cyano-4-hydroxycinnamic acid (α-CHCA) was purchased from TCI America. Potassium chloride (KCl) was purchased from Macron. Sodium chloride (NaCl) was purchased from EMD. Sodium azide ($NaN_3$) was purchased from Amresco. All reactions were conducted in 8 mL Chemglass reaction vials with TFE lined silicone SURE-LINK septa caps. Reactions were evacuated with ultra-high purity nitrogen from Airgas using a Schlenk line connected to purging needles.

### General Synthesis

4-dimethylaminopyridine (DMAP) and D,L-lactide (L) were weighed into 8 mL reaction vials. Propargyl alcohol (PA) or α-methyl propargyl alcohol (αMPA) was added volumetrically. Ratios of PA:L:DMAP or αMPA:L:DMAP that were investigated are listed in the text and in Table S1. A stir bar was added to the vial, and reactions were purged with nitrogen for 20 minutes. Vials were submerged in preheated 130 °C oil for times listed in the text and Table S1. After polymerizations, reaction vials were removed from oil, opened to atmosphere, and cooled before the slow addition of 1 mL of DCM. Reactions were recapped, vortexed until dissolved, and precipitated into 14 mL of hexanes in 15 mL conical tubes. Reactions were recapped, vortexed until dissolved, and precipitated into 14 mL of hexanes in 15 mL conical tubes. Tubes were shaken to aid precipitation, and supernatant was decanted. Fresh 15 mL of hexanes was added and shaken, and products were collected by decanting the supernatant and drying under an air stream. Samples were placed under vacuum to remove residual volatile solvents.

### Hydroxyl end group modification

After lactide polymerization or Huisgen 1,3-dipolar cycloaddition, reaction vials were removed from oil and opened to atmosphere, and succinic anhydride (SA) was added in a ratio of 1:1 initiator:SA. Vials were recapped and stirred at 130 °C for 5 minutes. Products were dissolved in DCM and precipitated in hexanes as described above.

### Huisgen 1,3-dipolar cycloaddition

A combination of 1.13 g 3-chloro-1-propanol and 1.17 g $NaN_3$ in 5 mL $ddH_2O$ was stirred at 80 °C for 16 hours. The product, 3-azido-1-propanol, was extracted with diethyl ether 10x using 5 mL per extraction. Combined extractions were dried with anhydrous sodium sulfate and filtered. Solvent was removed via rotary evaporation. Successful substitution reaction was confirmed by peak shifts in $^1$H-NMR spectra, and the yield was 93% (Fig. S20).

Azide-alkyne cycloaddition was conducted by dissolving ~200 mg of precipitated propargyl-functional oligo(lactide) in DMF and adding ~30 mg (2x excess) 3-azido-1-propanol. The mixture was stirred at 92 °C for 17 hours and precipitated in a mixture of 15 mL diethyl ether and 35 mL hexanes. Tubes were shaken to aid precipitation, and supernatant was decanted. Samples were placed under vacuum to remove residual volatile solvents.

### $^1$H-NMR

Samples were dissolved in deuterated chloroform without TMS and analyzed by 1D $^1$H-NMR (Brüker 400 MHz or 500 MHz, $CDCl_3$, 25 °C, 16 scans). Spectra for publication were prepared using Mestrelab Research MNova 11.

### Matrix assisted laser desorption ionization time of flight (MALDI-TOF)

Samples and α-CHCA were prepared at 10 mg/mL using 1:1 acetonitrile:dd$H_2O$ with 0.1% TFA. NaCl and KCl were prepared at 100 mg/mL in distilled, deionized water. Solutions were combined 10:10:1 (sample:α-CHCA:salt) and spotted on a Brüker MSP 96 target ground steel plate using 1 µL. Calibration was performed using aliquoted peptide calibration standards (Sigma-Aldrich). Linear mode suppressing ions below 200 Da exhibited the greatest signal/noise in spectra and was used for analysis. Data presented for publication were exported from Brüker Flex Analysis software, compiled using MATLAB R2016a, and graphed using GraphPad Prism 6.

### Peak designation by MALDI-TOF analysis

Three samples (1:20:4 PA:L:DMAP, 1:20:4 αMPA:L:DMAP, and 20:4 L:DMAP) were analyzed with three sample preparations (no salt dopant, NaCl dopant, and KCl dopant) and compared (Figure S1). There were no differences between NaCl and KCl dopants, as well as no differences between salt dopant and no salt dopant. This suggested all species present were complexed with DMAP, as nitrogen-containing compounds are cationized by hydrogens in the solvent solution. Moreover, $^1$H-NMR analysis identified four distinct CH and two distinct $CH_3$ proton shifts for DMAP, suggesting the presence of both DMAP catalyst and DMAP adducts in products. All peaks with MALDI spectra fell into one of two categories: (1) those numerically identical, and (2) those numerically separated by 14 Da, the mass difference between PA and αMPA ($CH_2$), between PA-initiated and αMPA-initiated reactions. Some peaks within category (1) were also numerically identical to those present in the DMAP-initiated/catalyzed reaction. Peak sets were designated by first identifying peaks separated by 72 Da, the mass of half a lactide unit, and then identifying the smallest identifiable peak in the series. These data were used to plot mass vs. lactide units, and the y-intercept of each plot was used to identify the base of each peak set (Figure S2).

## Figures and Tables

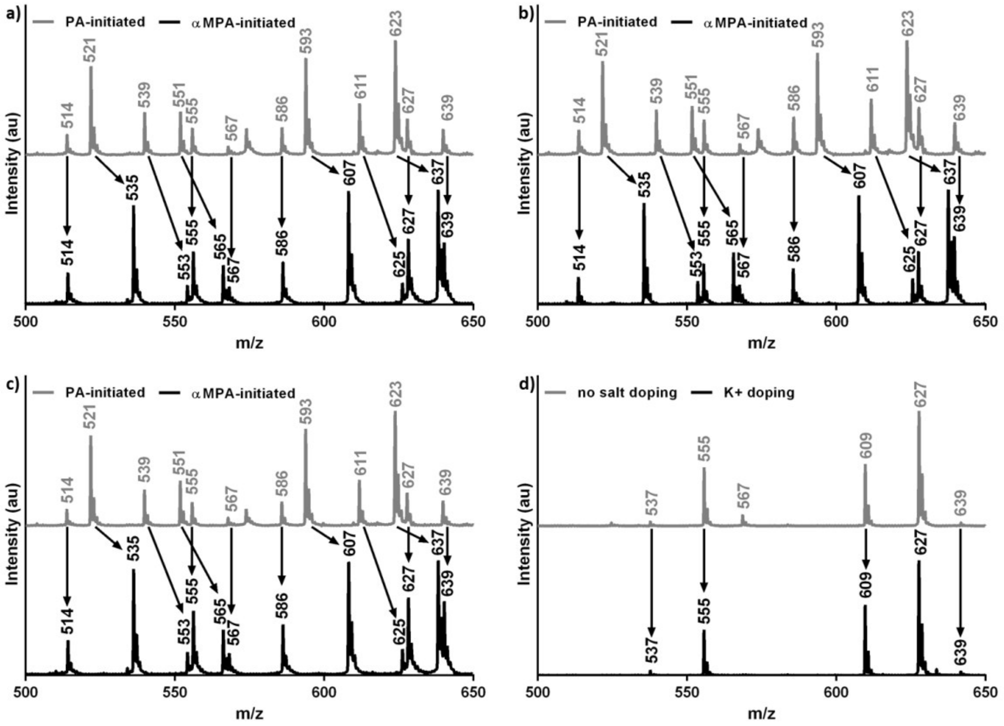


**Figure S1.**

(a-c) MALDI-TOF analysis of DMAP-catalyzed and propargyl alcohol-initiated (top, grey) or α-methyl propargyl alcohol-initiated (bottom, black) ROP of D,L-lactide prepared with (a) no salt doping, (b) NaCl salt doping, and (c) KCl salt doping. (d) DMAP-catalyzed/initiated ROP of D,L-lactide using either no salt doping (top, grey) or KCl salt doping (bottom, black).

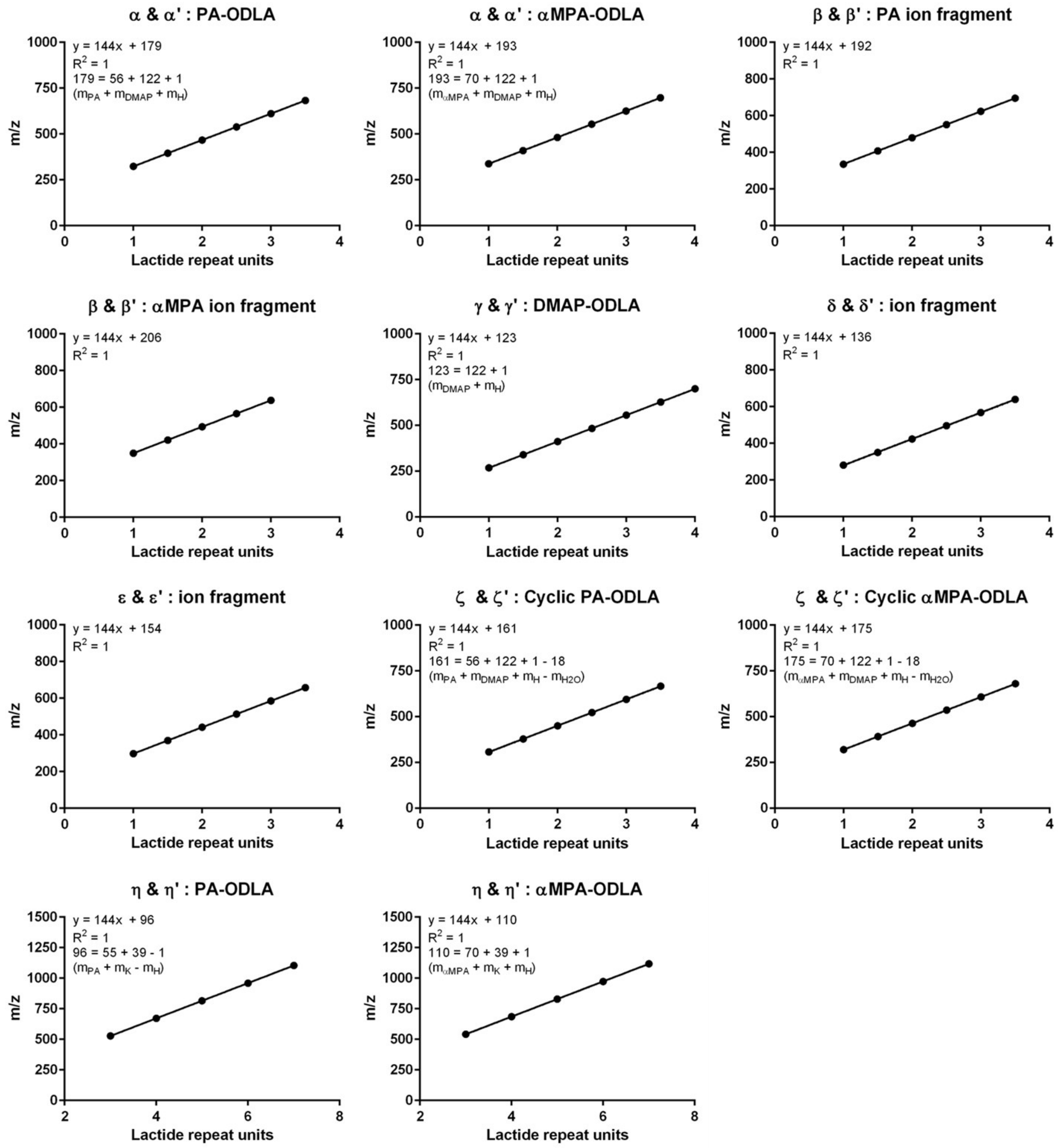


**Figure S2.**

Linear regression of peak sets to identify end groups of oligo(lactide) formed during polymerizations of propargyl alcohol (PA), 4-dimethylaminopyridine (DMAP), and D,L-lactide or α-methyl propargyl alcohol (αMPA), DMAP, and D,L-lactide.

**Table S1.**

Reagent ratios and polymerization times for all reactions described in the text.

| *Initiator* | *Ratio*[a] | *Time* | *% conv.*[b] | *$M_n$ by $^1$H-NMR*[c] | *$M_n$/$M_w$, PDI by MALDI-TOF*[d] | *Notes* |
|---|---|---|---|---|---|---|
| PA | 1:20:4 | 5 min | 99% | 2825 Da | 752/847 Da, 1.12 | |
| PA | 1:20:4 | 10 min | 97% | 2124 Da | 833/955 Da, 1.15 | |
| PA | 1:20:4 | 15 min | 98% | 2606 Da | 817/930 Da, 1.14 | |
| PA | 1:20:4 | 30 min | 97% | 2367 Da | 847/964 Da, 1.14 | |
| PA | 1:20:4 | 60 min | 98% | 2985 Da | 823/963 Da, 1.17 | |
| PA | 1:5:0.06 | 5 min | 72% | 759 Da | 660/732 Da, 1.11 | 10 wt% HQ |
| DMAP | 0:20:4 | 5 min | 80% | 3720 Da | 794/895 Da, 1.13 | DMAP-initiated |
| PA | 1:20:0 | 5 min | 0% | N/A | N/A | No reaction |
| αMPA | 1:20:0 | 5 min | 0% | N/A | N/A | No reaction |
| αMPA | 1:20:4 | 5 min | 96% | 2041 Da | 721/840 Da, 1.16 | |
| αMPA | 1:20:4 | 10 min | 96% | 1922 Da | 779/890 Da, 1.14 | |
| αMPA | 1:20:4 | 15 min | 97% | 2036 Da | 822/919 Da, 1.12 | |
| αMPA | 1:20:4 | 30 min | 97% | 2127 Da | 856/956 Da, 1.12 | |
| αMPA | 1:20:4 | 60 min | 97% | 2104 Da | 850/947 Da, 1.11 | |
| αMPA | 1:20:2 | 5 min | 87% | 3620 Da | 782/938 Da, 1.20 | |
| αMPA | 1:20:1 | 5 min | 88% | 3150 Da | 826/1017 Da, 1.23 | |
| αMPA | 1:20:0.5 | 5 min | 65% | 3120 Da | 881/1087 Da, 1.23 | |
| αMPA | 1:10:1 | 5 min | 92% | 2200 Da | 759/947 Da, 1.25 | |
| αMPA | 1:5:1 | 5 min | 97% | 1470 Da | 763/883 Da, 1.16 | |
| αMPA | 1:2:1 | 5 min | 97% | 1160 Da | 656/719 Da, 1.10 | |
| αMPA | 1:10:5 | 5 min | 96% | 2050 Da | 772/858 Da, 1.11 | |
| αMPA | 1:5:2.5 | 5 min | 97% | 1550 Da | 725/809 Da, 1.12 | |
| αMPA | 1:2:0.03 | 5 min | 82% | 480 Da | 619/701 Da, 1.13 | |
| αMPA | 1:2:0.03 | 10 min | 99% | 690 Da | 685/764 Da, 1.11 | |
| αMPA | 1:2:0.03 | 15 min | 99% | 760 Da | 661/734 Da, 1.11 | |
| αMPA | 1:5:0.06 | 5 min | 73% | 720 Da | 753/873 Da, 1.16 | |
| αMPA | 1:5:0.06 | 10 min | 89% | 880 Da | 727/829 Da, 1.14 | |
| αMPA | 1:5:0.06 | 15 min | 94% | 1020 Da | 758/870 Da, 1.15 | |
| αMPA | 1:10:0.11 | 5 min | 32% | 1010 Da | 772/953 Da, 1.23 | |
| αMPA | 1:10:0.11 | 10 min | 71% | 1280 Da | 736/857 Da, 1.16 | |
| αMPA | 1:10:0.11 | 15 min | 83% | 1460 Da | 747/896 Da, 1.20 | |
| αMPA | 1:2:0.03 | 1 min | 0% | N/A | N/A | No reaction |
| αMPA | 1:2:0.03 | 2 min | 68% | 942 Da | 631/711 Da, 1.12 | |
| αMPA | 1:2:0.03 | 3 min | 91% | 991 Da | 647/725 Da, 1.12 | |
| αMPA | 1:2:0.03 | 4 min | 90% | 1617 Da | 686/765 Da, 1.12 | |
| αMPA | 1:2:0.03 | 90 sec | 62% | 489 Da | 668/756 Da, 1.13 | |

[a]Ratios are PA:L:DMAP or αMPA:L:DMAP. [b]Percent conversion (X) calculated by $^1$H-NMR via peak integrals indicated in Fig. 1a. X = 100*area(peak C)/[area(peak C)+area(peak D)]. [c]$M_n$ = [area(peak C)+area(peak F)]*144+56 for PA-initiated; [area(peak C)+area(peak F)]*72+70 for αMPA. [d]$M_n = \Sigma N_i M_i / \Sigma N_i$ and $M_w = \Sigma N_i M_i^2 / \Sigma N_i M_i$, where $N_i$ is the intensity of the "$i^{th}$" peak, and $M_i$ is the mass of the "$i^{th}$" peak. PDI = $M_w$/$M_n$.

a)

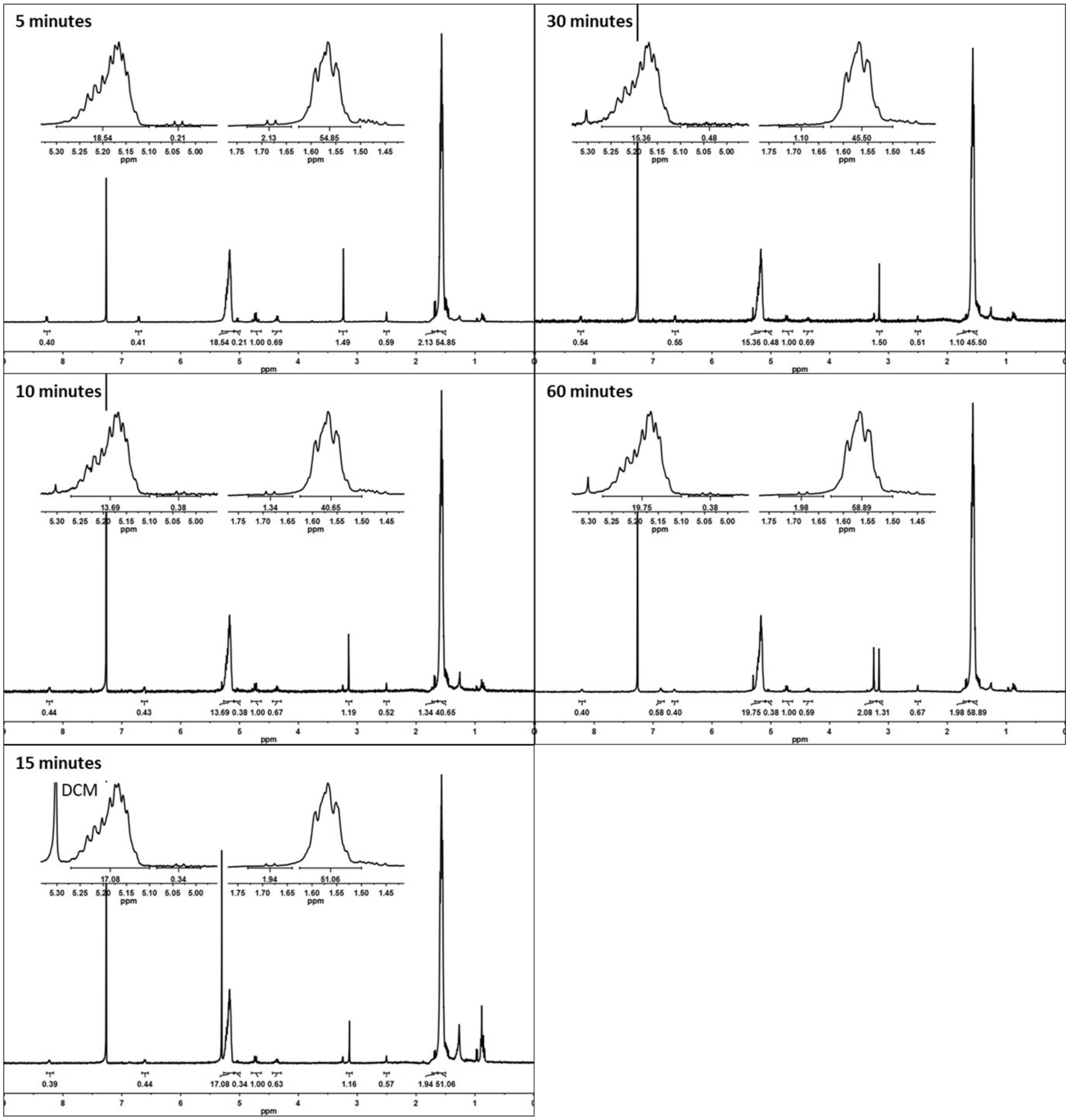


**Figure S3a.**

$^{1}$H-NMR for polymerizations of 1:20:4 propargyl alcohol:lactide:4-dimethylaminopyridine (PA:L:DMAP) reacted for 5-60 minutes. DCM, dichloromethane.

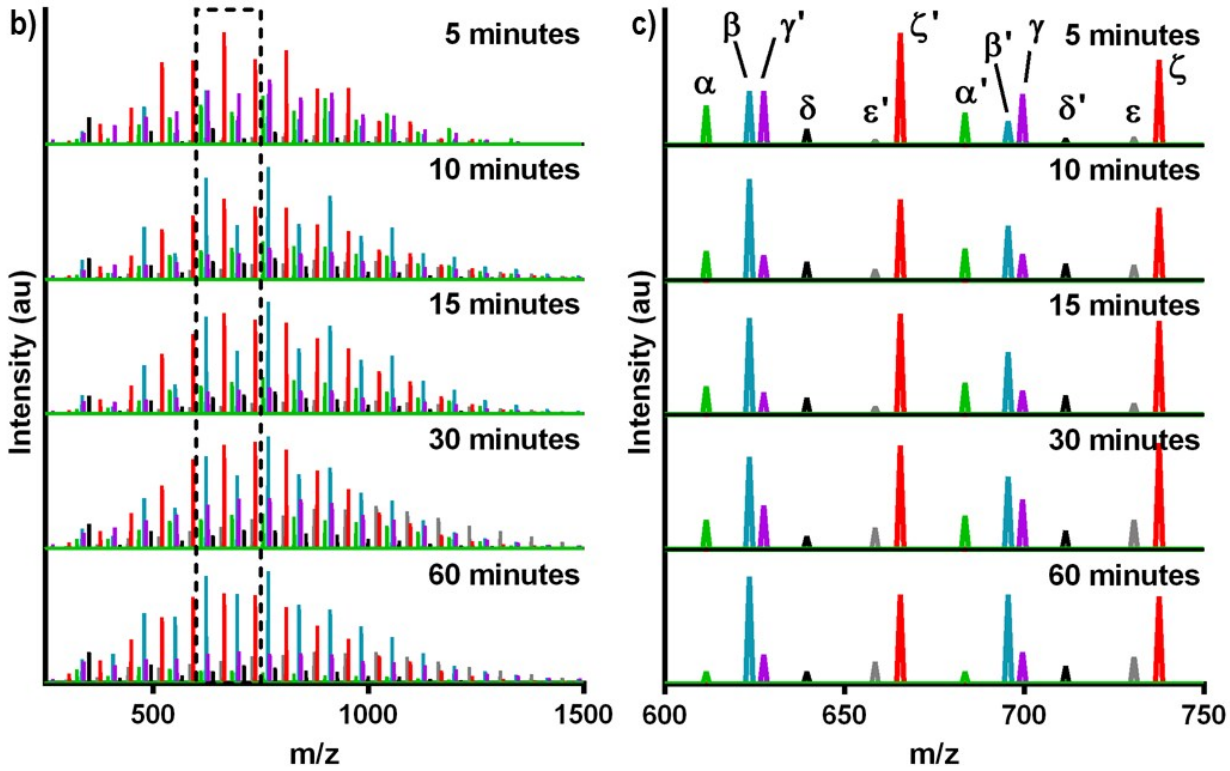


**Figure S3b-c.**

MALDI-TOF for polymerizations of 1:20:4 propargyl alcohol:lactide:4-dimethylaminopyridine (PA:L:DMAP) reacted for 5-60 minutes, where (c) is one 144 Da peak set of (b).

a)

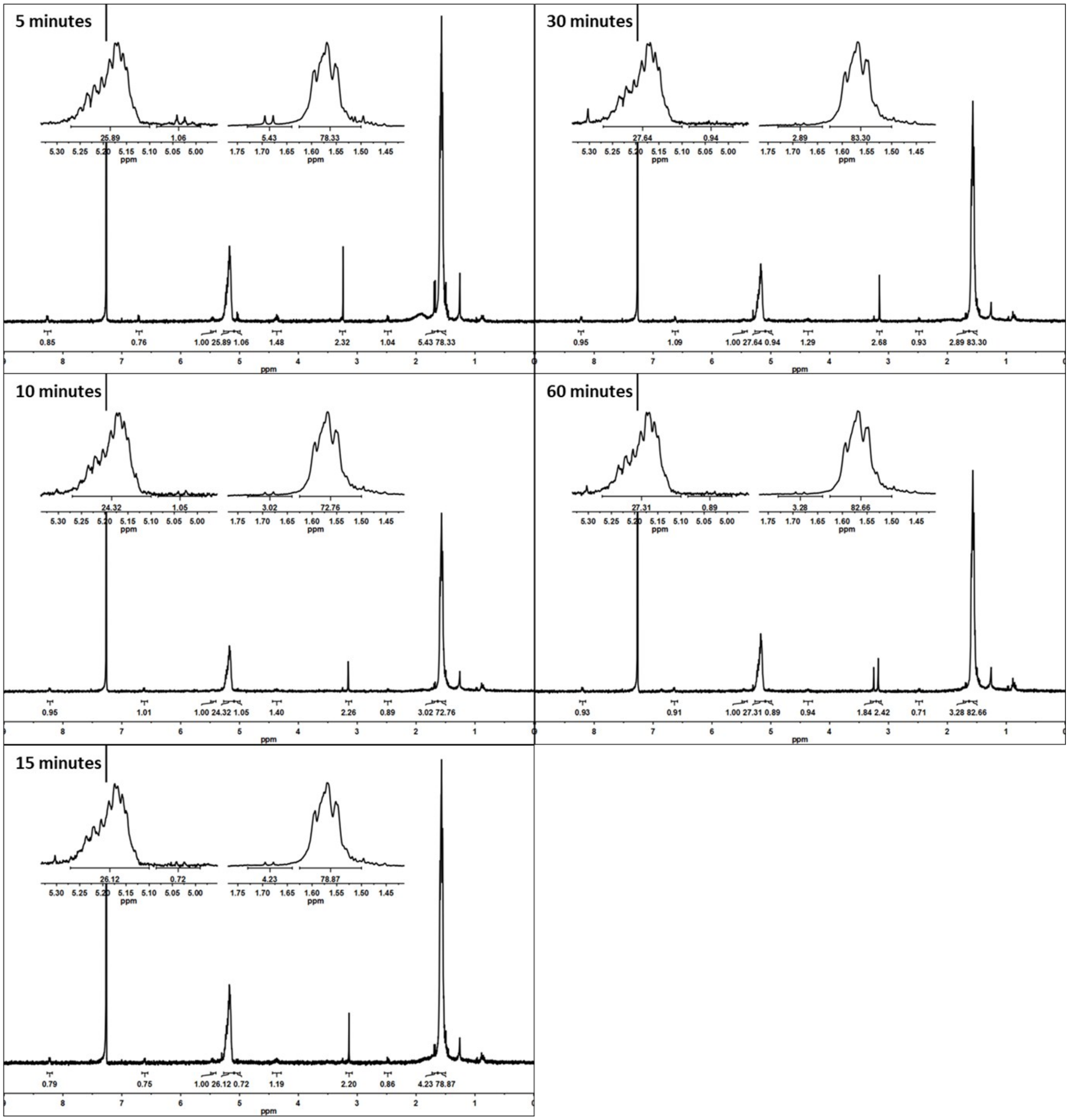


**Figure S4a.**

$^{1}$H-NMR for polymerizations of 1:20:4 α-methyl propargyl alcohol:lactide:4-dimethylaminopyridine (αMPA:L:DMAP) reacted for 5-60 minutes**.**

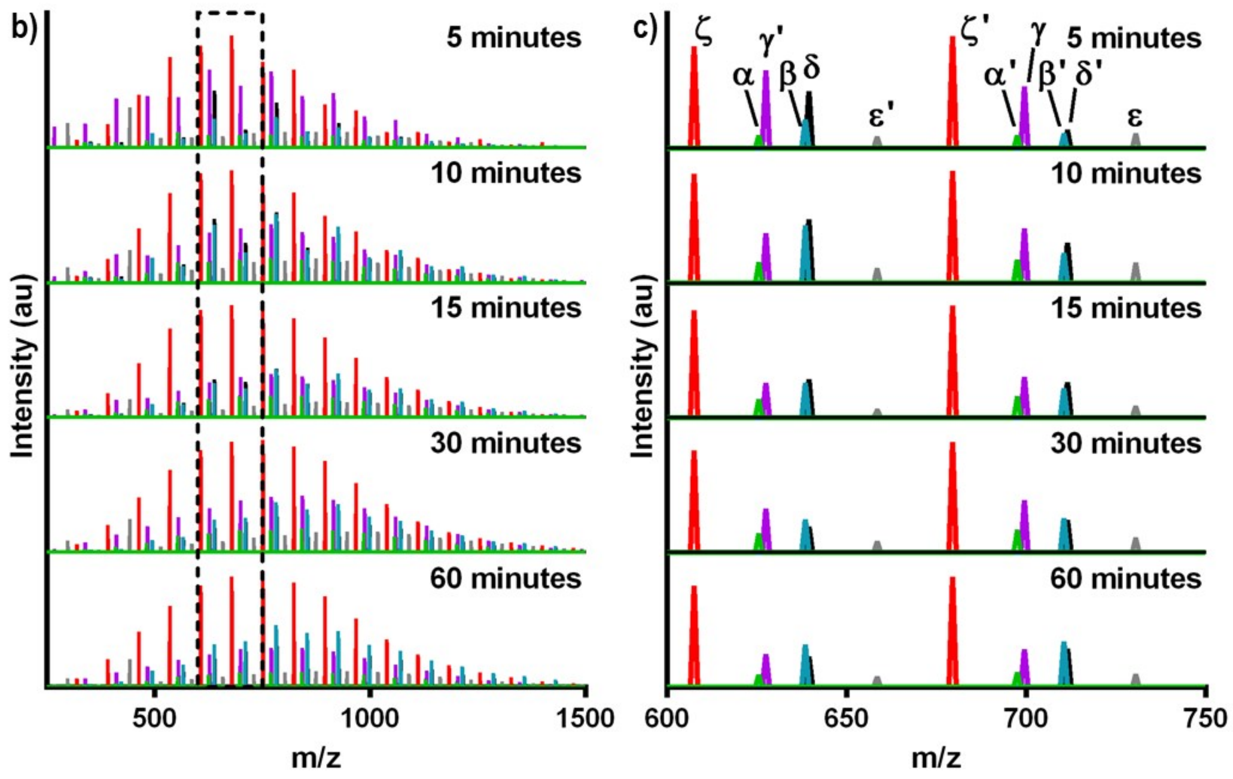


**Figure S4b-c.**

MALDI-TOF for polymerizations of 1:20:4 α-methyl propargyl alcohol:lactide:4-dimethylaminopyridine (αMPA:L:DMAP) reacted for 5-60 minutes, where (c) is one 144 Da peak set of (b).

## Table S2.

Peak identities for oligo(D,L-lactide) (ODLA) presented in Fig. 1, Fig. S3, and Fig. S4.

| ***Peak*** | ***Identity*** | ***Structure*** |
|---|---|---|
| **α** | PA-ODLA-DMAP-H$^+$, n odd<br>αMPA-ODLA-DMAP-H$^+$, n odd | 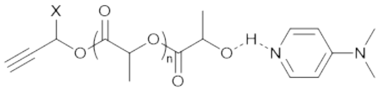<br>X = H, propargyl alcohol; $CH_3$, α-methyl propargyl alcohol. |
| **α'** | PA-ODLA-DMAP-H$^+$, n even<br>αMPA-ODLA-DMAP-H$^+$, n even | |
| **γ** | DMAP-ODLA-H$^+$, n odd | 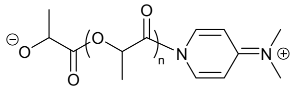 |
| **γ'** | DMAP-ODLA-H$^+$, n even | |
| **ζ** | Cyclic PA-ODLA, n+m odd<br>Cyclic αMPA-ODLA, n+m odd | 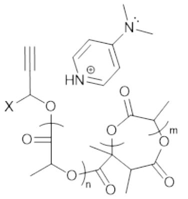<br>X = H, propargyl alcohol; $CH_3$, α-methyl propargyl alcohol. |
| **ζ'** | Cyclic PA-ODLA, n+m even<br>Cyclic αMPA-ODLA, n+m even | |
| **η** | αMPA-ODLA-K$^+$, n odd | 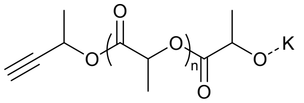 |
| Fig. S8 | HQ-DMAP-ODLA-H$^+$ | Undefined |
| Fig. S10 | Cyclic DMAP-ODLA, n+m odd | 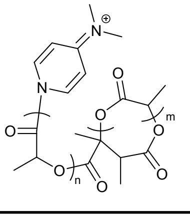 |
| | Cyclic DMAP-ODLA, n+m even | |

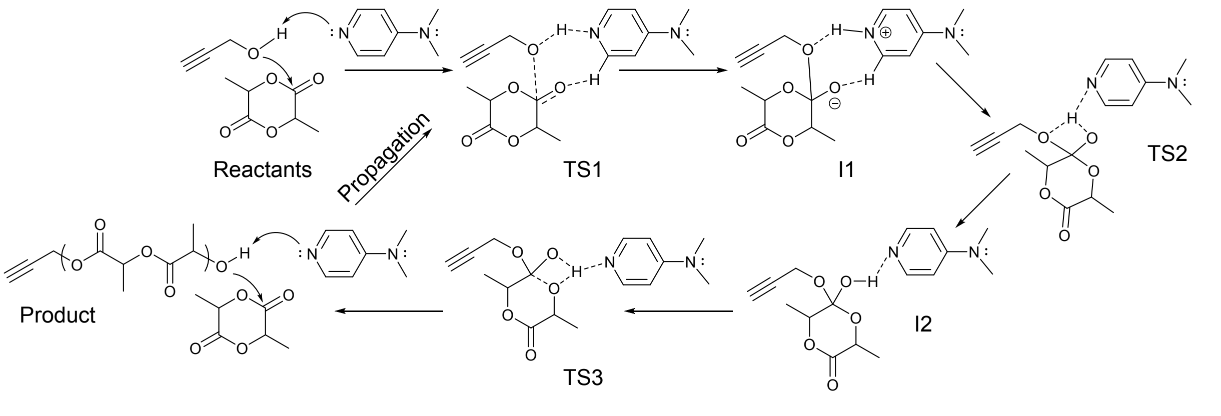

**Figure S5.**

Base activation of propargyl alcohol by 4-dimethylaminopyridine to initiate ring-opening polymerization of D,L-lactide.

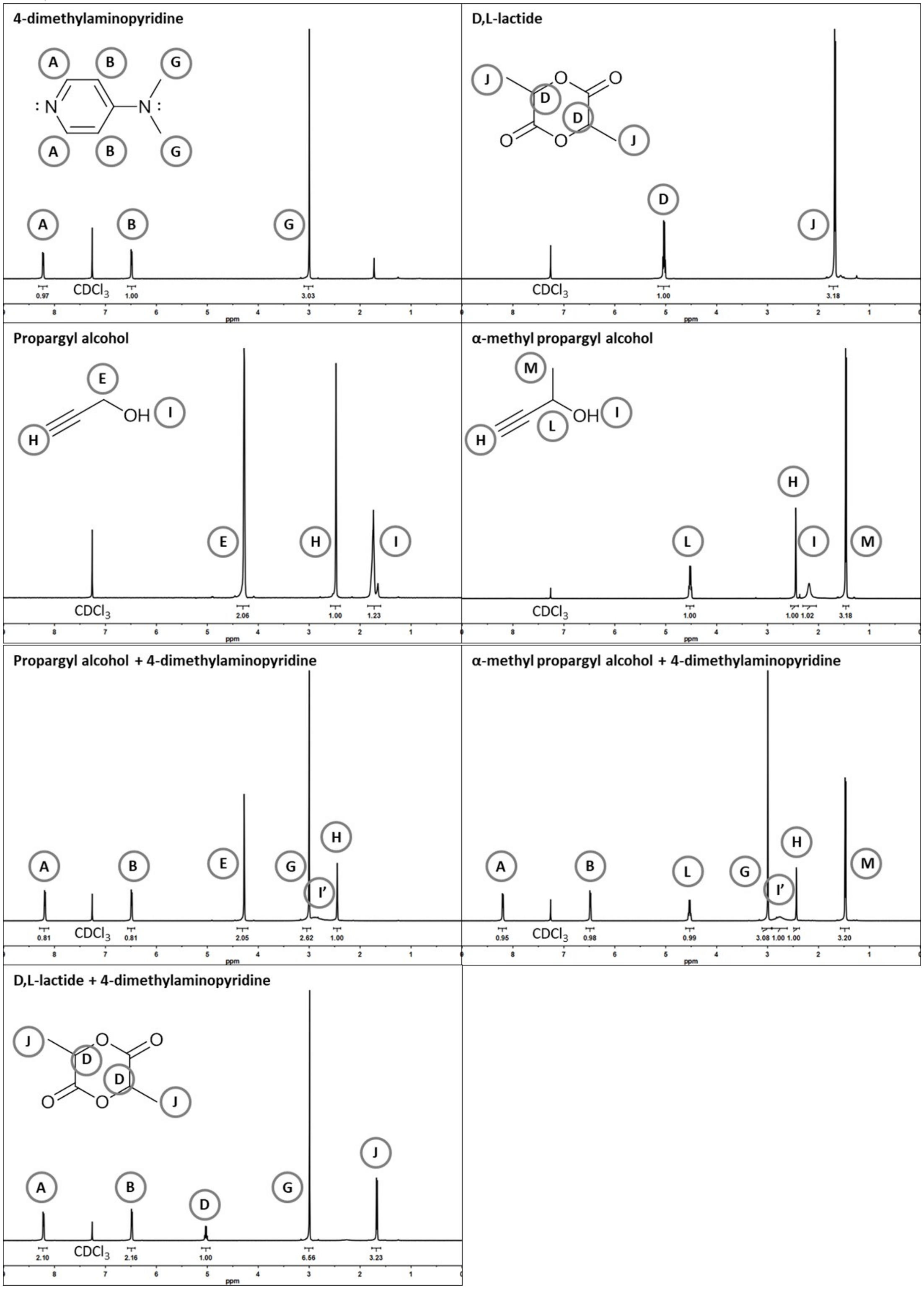

**Figure S6.**

$^{1}$H-NMR of 4-dimethylaminopyridine (DMAP), D,L-lactide, propargyl alcohol (PA), and α-methyl propargyl alcohol (αMPA), and combinations of DMAP and PA, DMAP and αMPA, and DMAP and lactide demonstrating that DMAP interacts with either alcohol but not lactide to initiate polymerization.

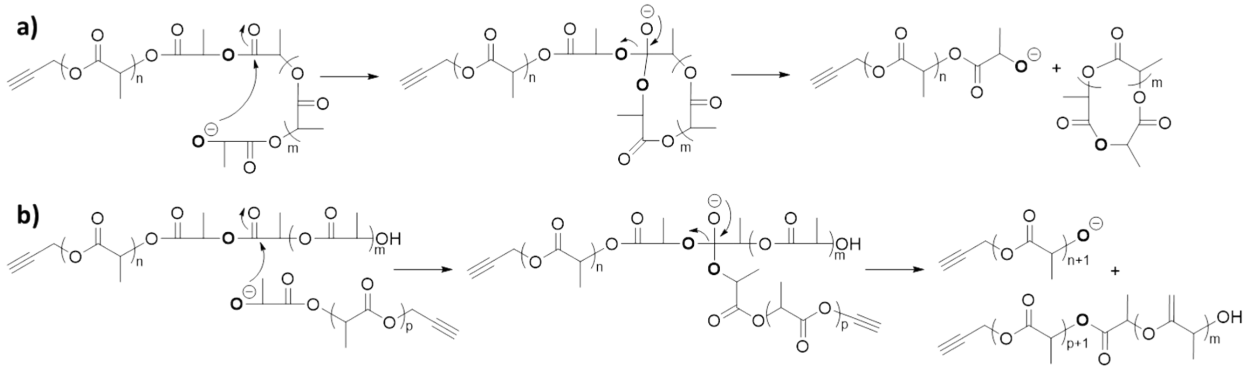


**Figure S7.**

Reaction mechanisms for (a) intramolecular and (b) intermolecular transesterification.

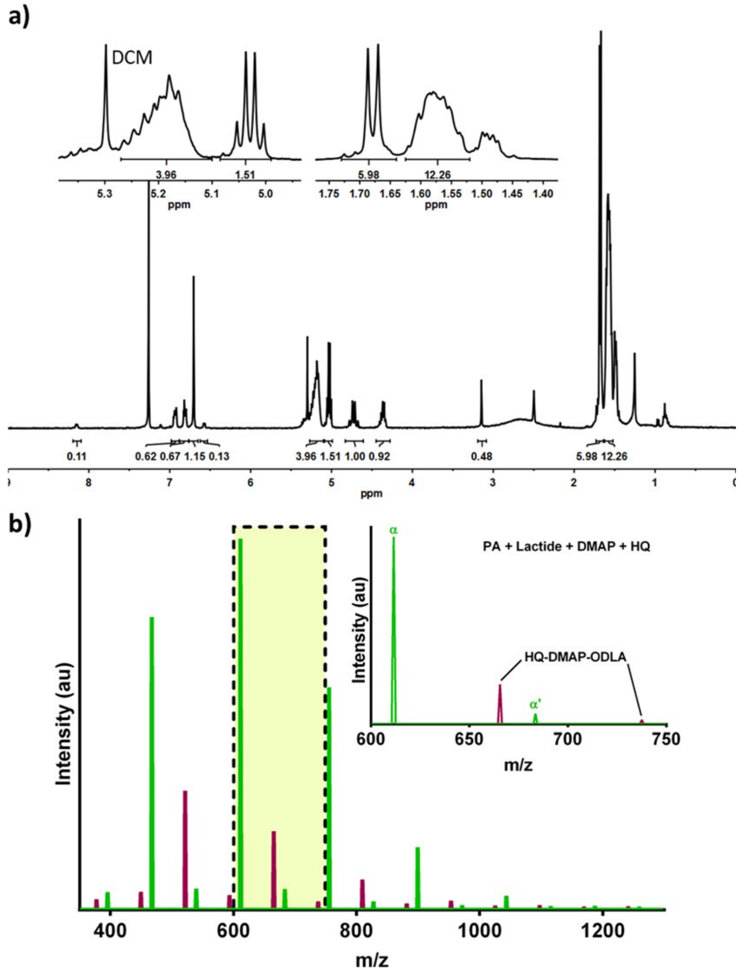

**Figure S8.**

Hydroquinone (HQ) was added to a polymerization of lactide with propargyl alcohol (PA) and 4-dimethylaminopyridine (DMAP) to investigate the effect on PA-ODLA cyclicization. (a) $^{1}$H-NMR demonstrates polymerization to ~72% conversion after 5 minutes. (b) MALDI-TOF shows the appearance of HQ-lactide-DMAP peaks (spectrum collected in reflector mode).

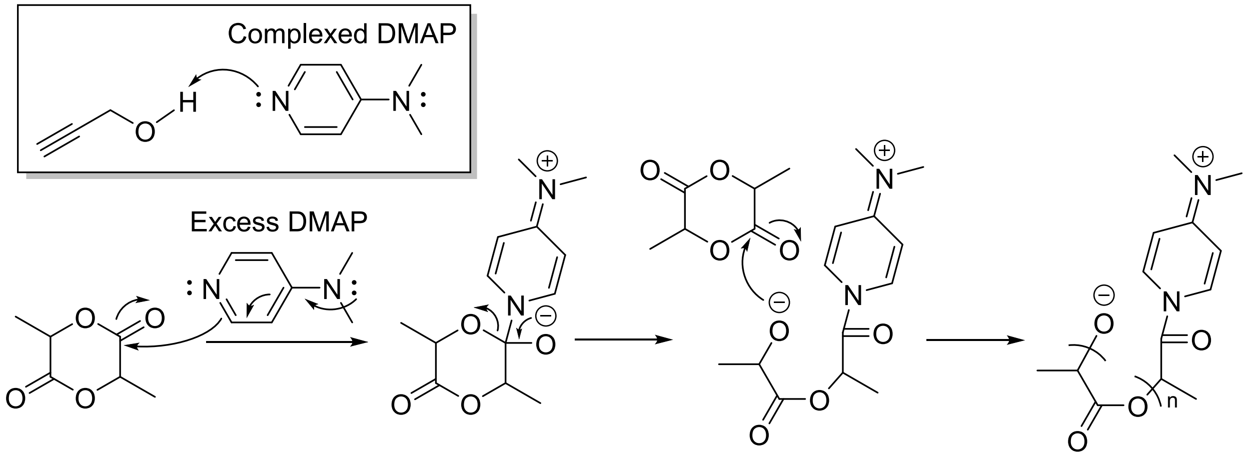


**Figure S9.**

Nucleophilic attack of 4-dimethylaminopyridine (DMAP) on lactide to initiate ring-opening polymerization when DMAP is available in excess relative to propargyl alcohol.

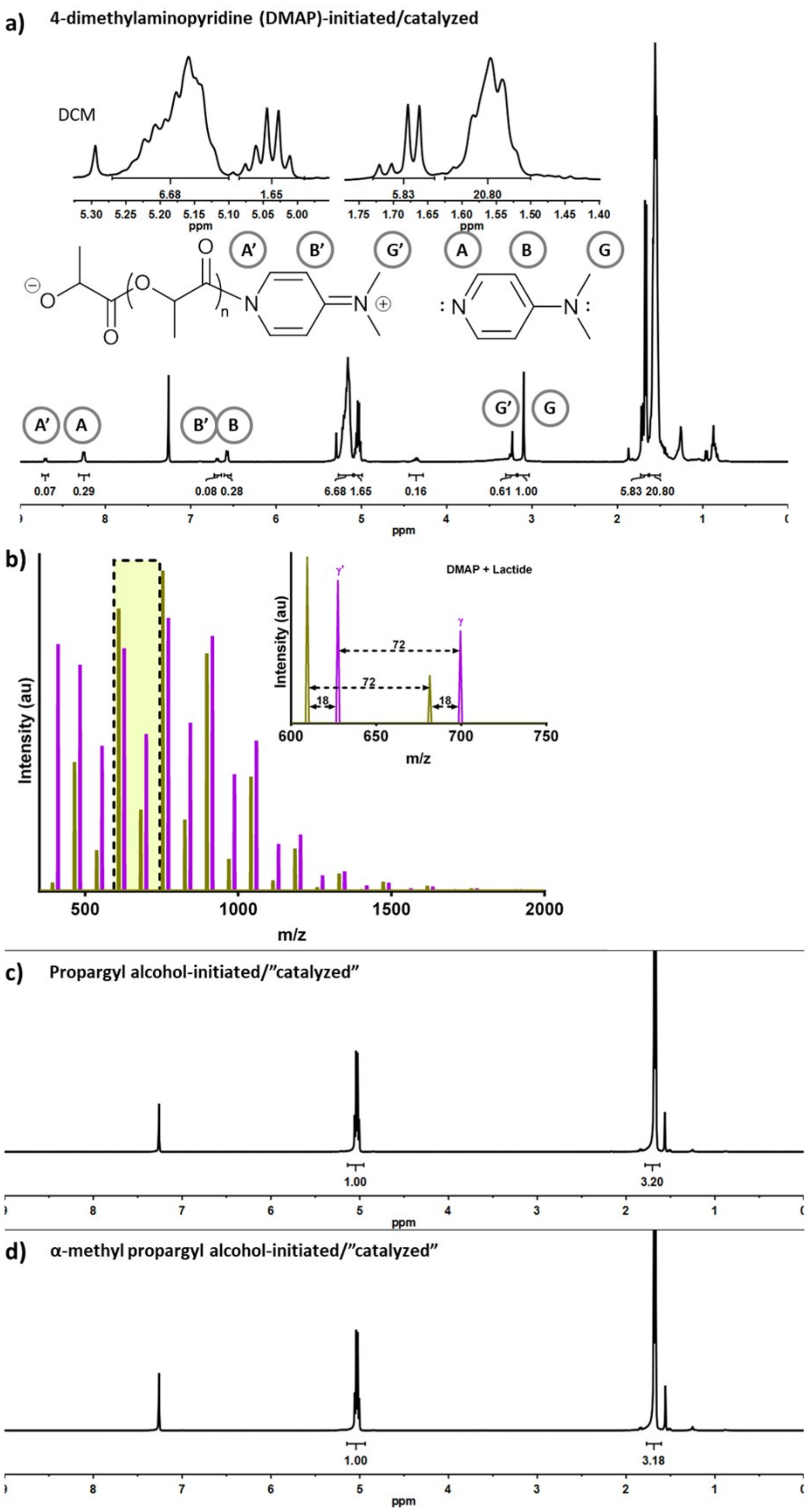


**Figure S10.**

(a) $^1$H-NMR and (b) MALDI-TOF demonstrate 4-dimethylaminopyridine (DMAP) can initiate and catalyze lactide polymerization (spectrum collected in reflector mode). (c) Propargyl alcohol and (d) α-methyl propargyl alcohol cannot self-initiate and catalyze, as only pure lactide monomer precipitates after "polymerization."

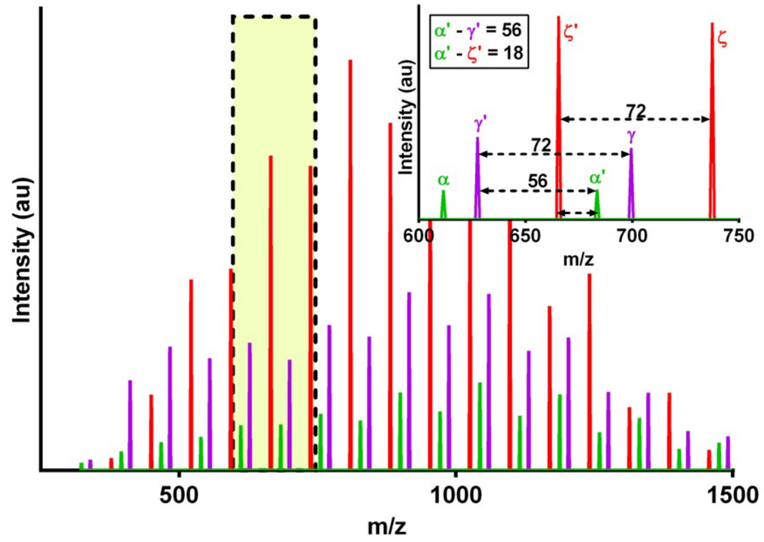


**Figure S11.**

MALDI-TOF of 5-minute 1:20:4 propargyl alcohol:lactide:4-dimethylaminopyridine (PA:L:DMAP) using reflector mode suggests that peaks sets “β,” “δ,” and “ε” are temporary ion fragments created during analysis and are not formed during polymerization (compare to Fig. 1b and Fig. S3b-c).

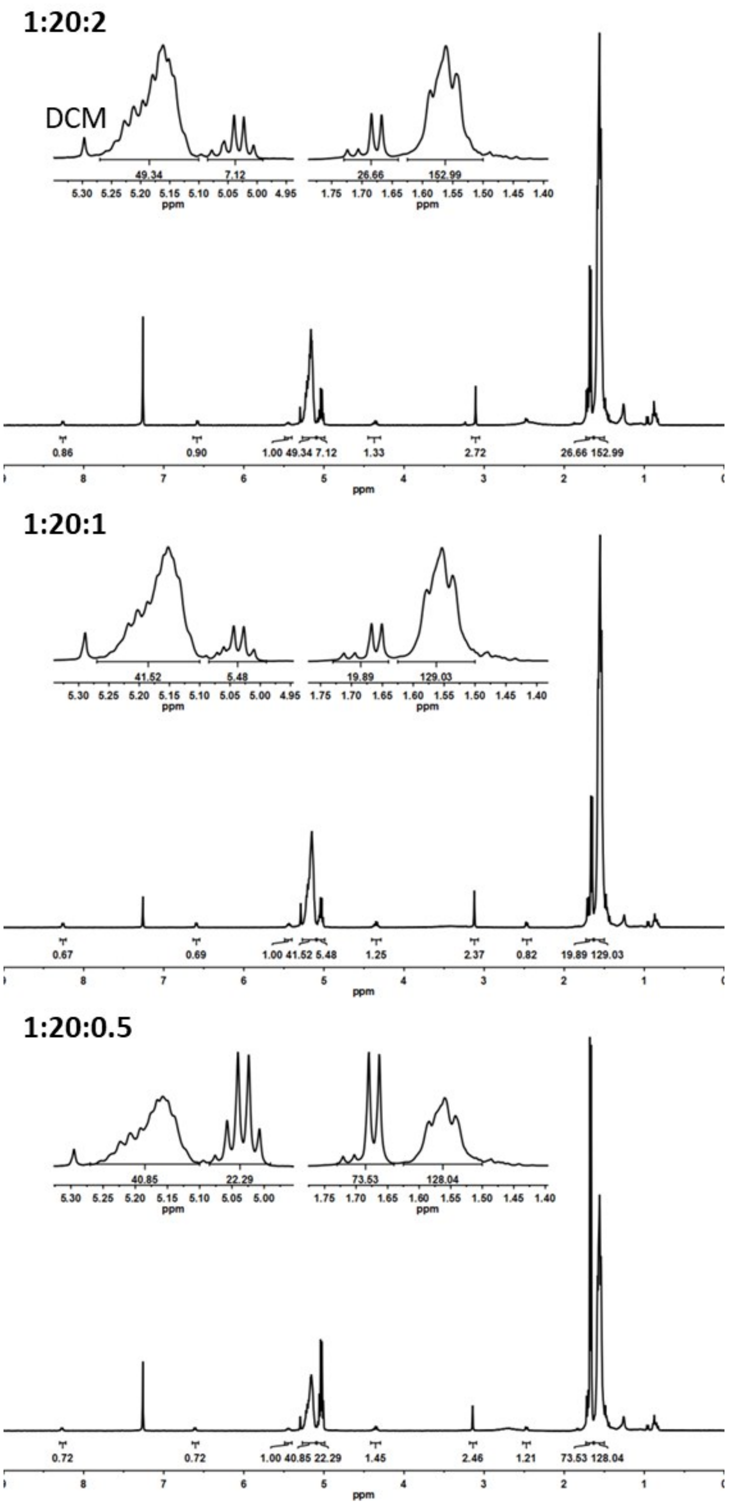


**Figure S12.**

$^{1}$H-NMR for polymerizations of 1:20:2, 1:20:1, and 1:20:0.5 α-methyl propargyl alcohol:lactide:4-dimethylaminopyridine (αMPA:L:DMAP) reacted for 5 minutes**.**

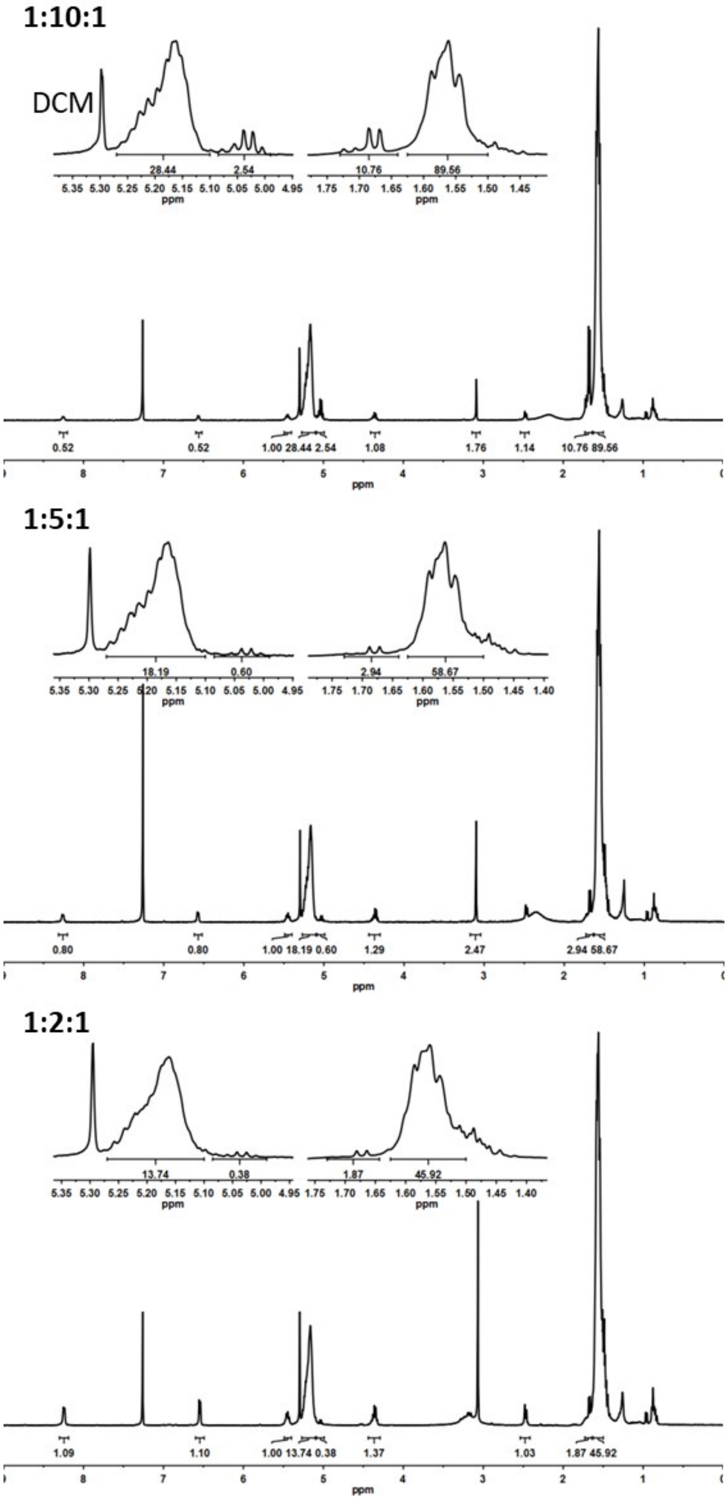


**Figure S13.**

$^1$H-NMR for polymerizations of 1:10:1, 1:5:1, and 1:2:1 α-methyl propargyl alcohol:lactide:4-dimethylaminopyridine (αMPA:L:DMAP) reacted for 5 minutes**.**

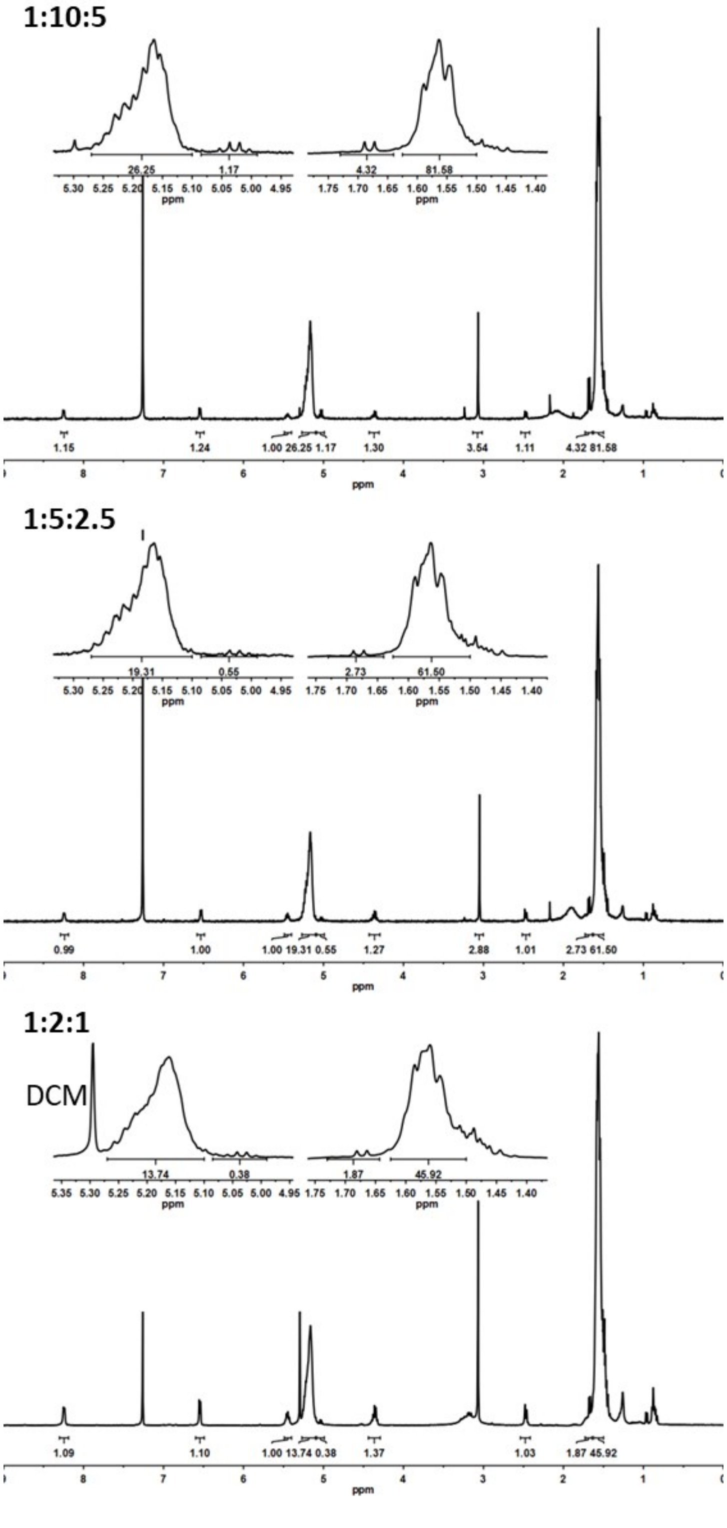


**Figure S14.**

$^1$H-NMR for polymerizations of 1:10:5, 1:5:2.5, and 1:2:1 α-methyl propargyl alcohol:lactide:4-dimethylaminopyridine (αMPA:L:DMAP) reacted for 5 minutes**.**

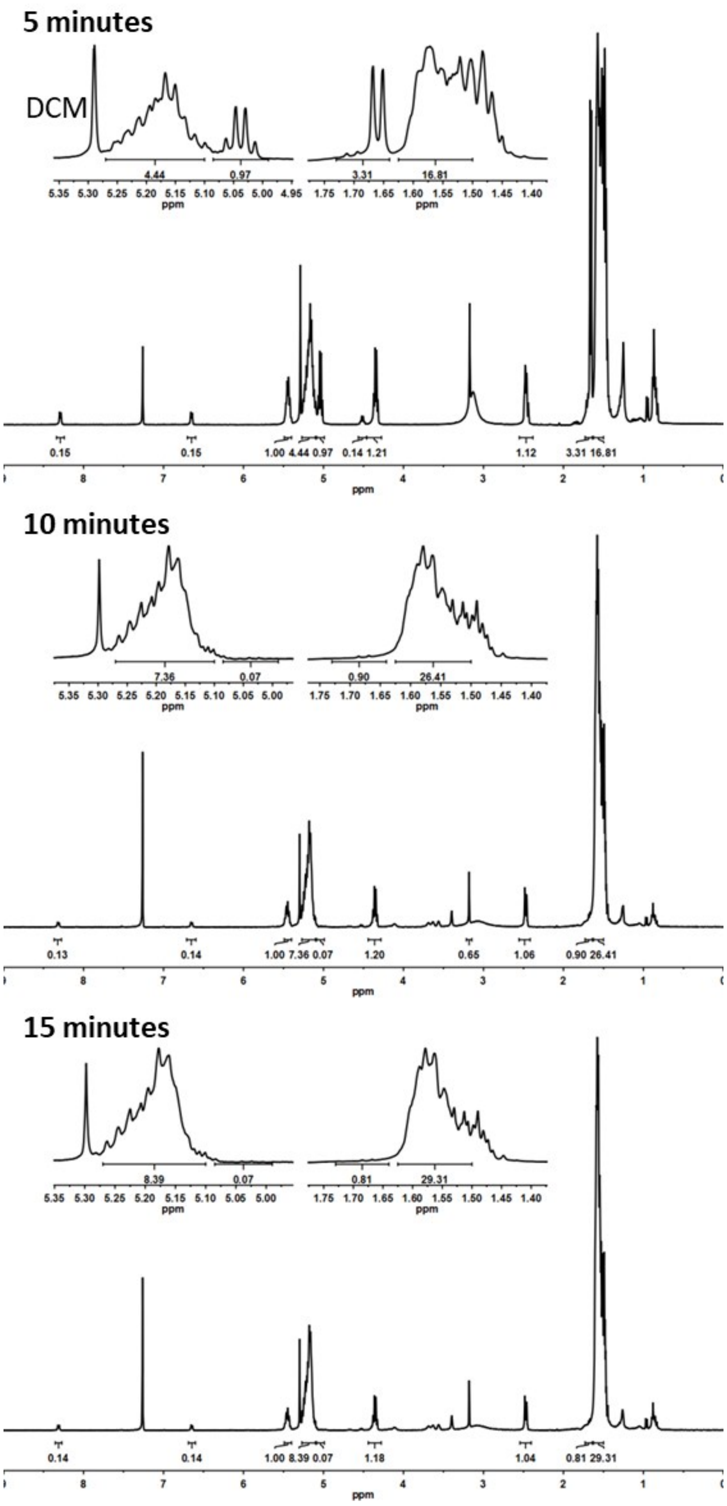


**Figure S15.**

$^{1}$H-NMR for polymerizations of 1:2:0.03 α-methyl propargyl alcohol:lactide:4-dimethylaminopyridine (αMPA:L:DMAP) reacted for 5, 10, and 15 minutes**.**

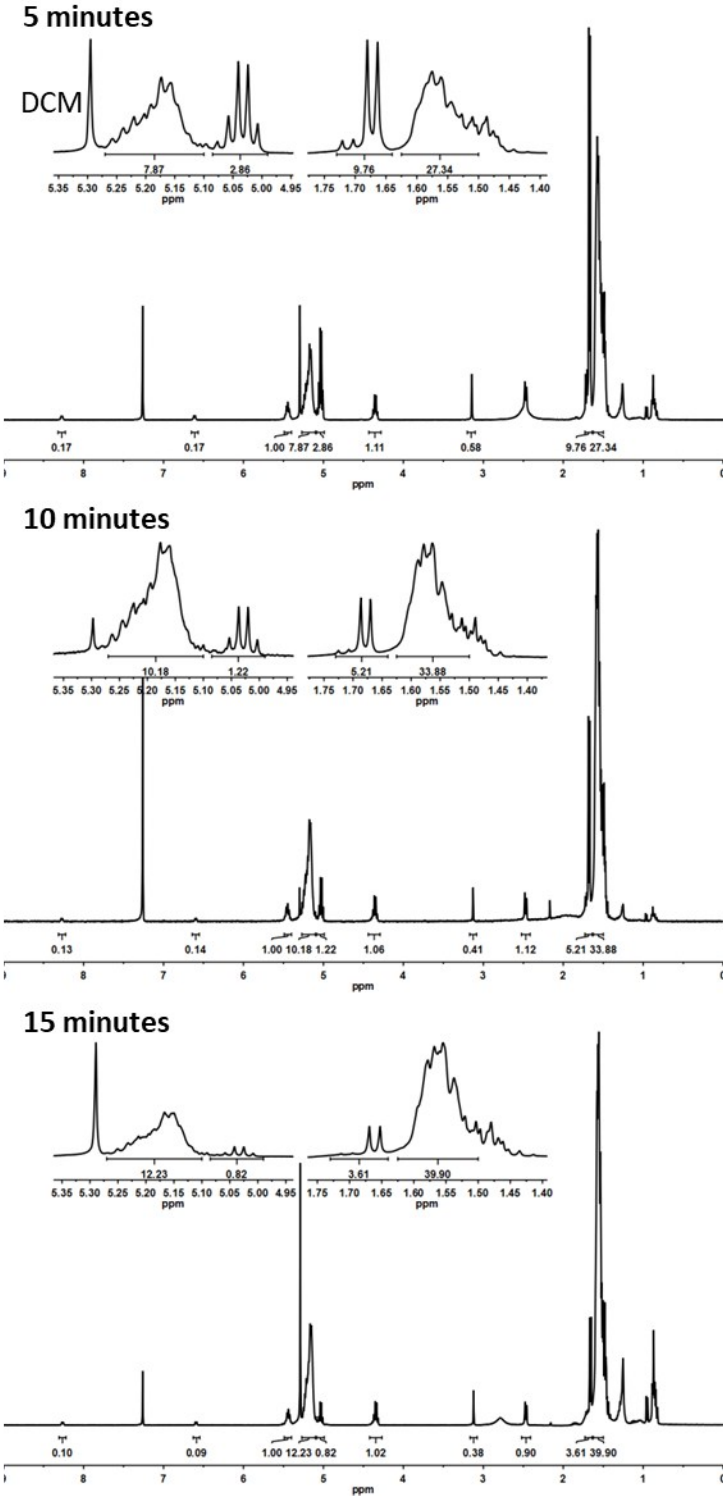


**Figure S16.**

$^1$H-NMR for polymerizations of 1:5:0.06 α-methyl propargyl alcohol:lactide:4-dimethylaminopyridine (αMPA:L:DMAP) reacted for 5, 10, and 15 minutes**.**

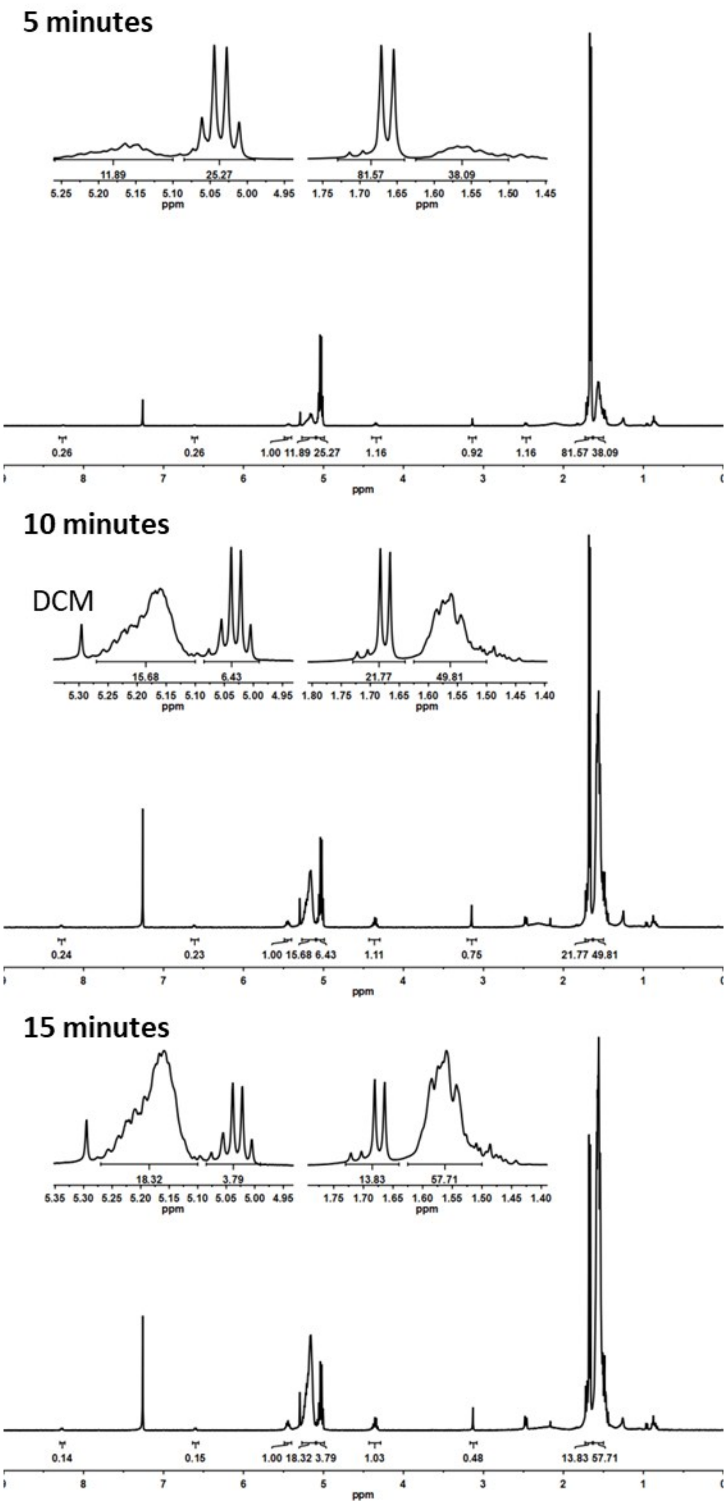


**Figure S17.**

$^{1}$H-NMR for polymerizations of 1:10:0.11 α-methyl propargyl alcohol:lactide:4-dimethylaminopyridine (αMPA:L:DMAP) reacted for 5, 10, and 15 minutes**.**

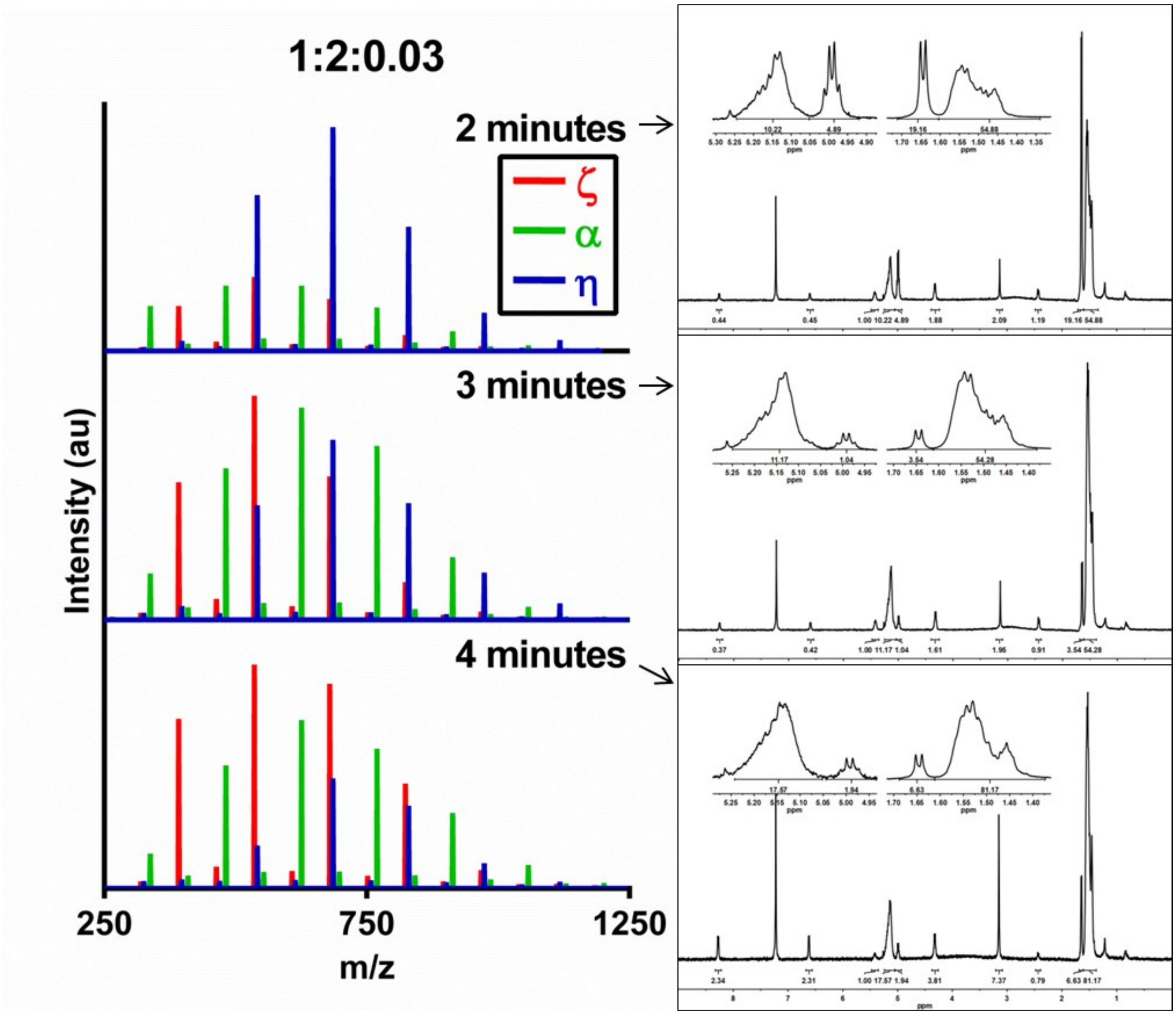


**Figure S18.**

MALDI-TOF and $^{1}$H-NMR for polymerizations of 1:2:0.03 α-methyl propargyl alcohol:lactide:4-dimethylaminopyridine (αMPA:L:DMAP) reacted for 2, 3, and 4 minutes**.**

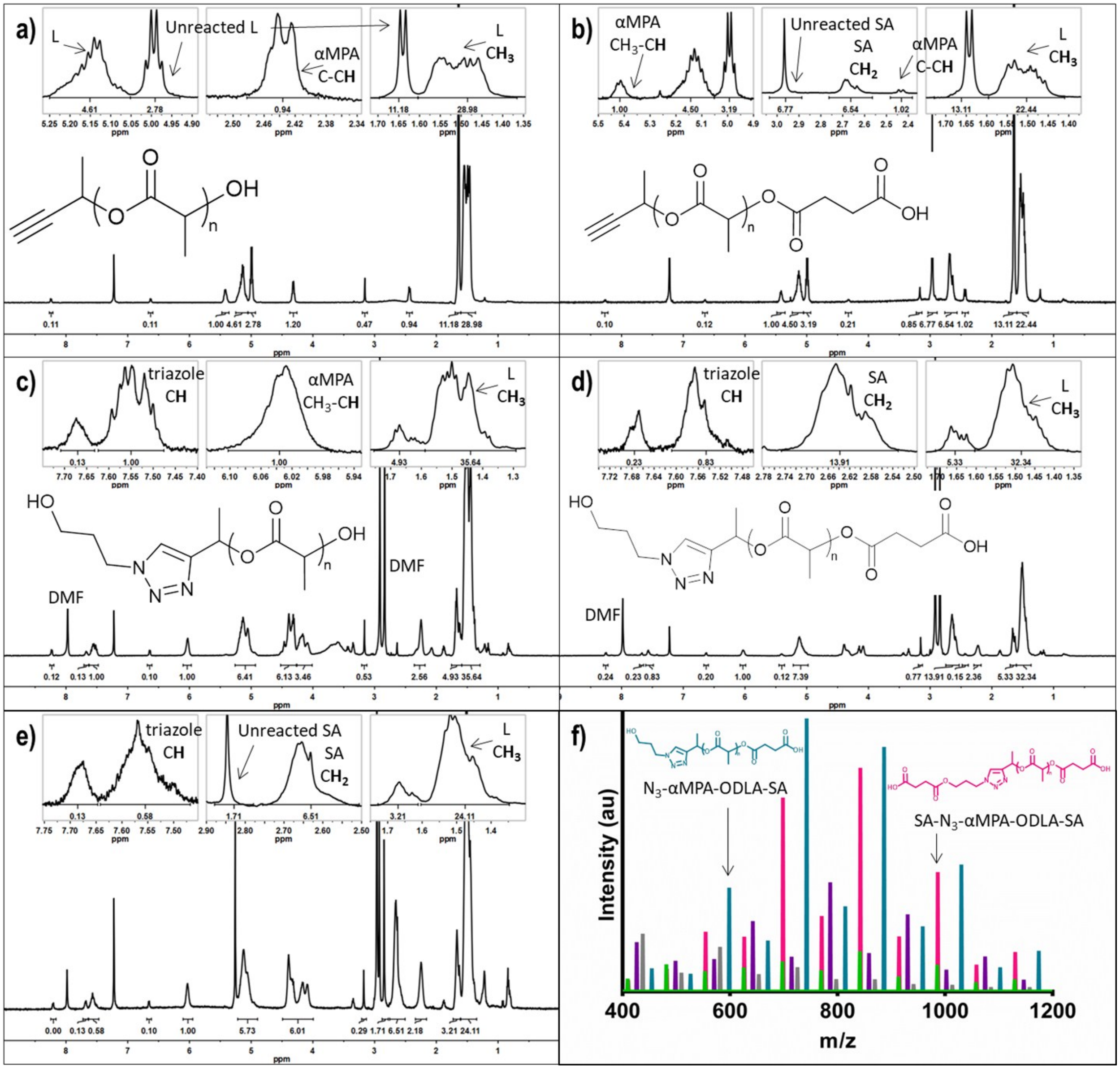

**Figure S19.**

$^{1}$H-NMR of proof of concept conjugations presented in Fig. 3 using (a) α-methyl propargyl alcohol-initiated oligo(D,L-lactide) (αMPA-ODLA) as a heterobifunctional linker. (b) αMPA-ODLA reacted with succinic anhydride (SA) to form αMPA-ODLA-SA. (c) 3-azido-1-propanol reacted with αMPA-ODLA to form $N_3$-αMPA-ODLA. (d) 3-azido-1-propanol reacted with αMPA-ODLA-SA to form $N_3$-αMPA-ODLA-SA. Note that SA-$N_3$-αMPA-ODLA-SA also forms. (e) SA reacted with $N_3$-αMPA-ODLA to form $N_3$-αMPA-ODLA-SA. Note that SA-$N_3$-αMPA-ODLA-SA also forms. (f) MALDI-TOF of (e).

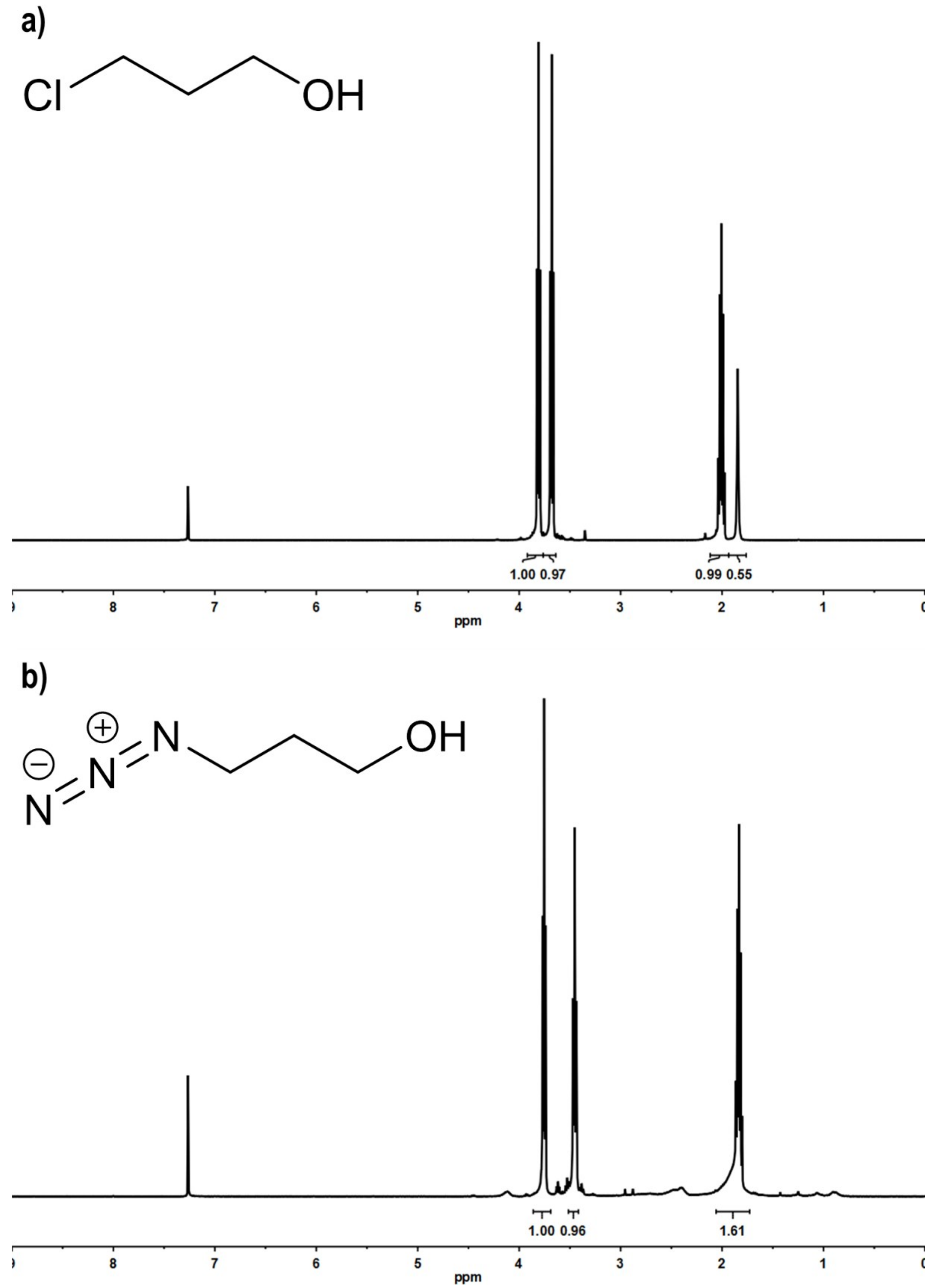


**Figure S20.**

$^{1}$H-NMR of 3-chloro-1-propanol precursor (a) and 3-azido-1-propanol product (b). Notice the ppm shifts of $CH_2$ peaks.
